# Nervous system–wide analysis of all *C. elegans* cadherins reveals neuron-specific functions across multiple anatomical scales

**DOI:** 10.1126/sciadv.ads2852

**Published:** 2025-02-21

**Authors:** Maryam Majeed, Chien-Po Liao, Oliver Hobert

**Affiliations:** Department of Biological Sciences, Howard Hughes Medical Institute, Columbia University, New York, NY, USA.

## Abstract

Differential expression of cell adhesion proteins is a hallmark of cell-type diversity across the animal kingdom. Gene family-wide characterization of their organismal expression and function is, however, lacking. Using genome-engineered reporter alleles, we established an atlas of expression of the entire set of 12 cadherin gene family members in the nematode *Caenorhabditis elegans*, revealing differential expression across neuronal classes, a dichotomy between broadly and narrowly expressed cadherins, and several context-dependent temporal transitions in expression across development. Engineered mutant null alleles of cadherins were analyzed for defects in morphology, behavior, neuronal soma positions, neurite neighborhood topology and fasciculation, and localization of synapses in many parts of the nervous system. This analysis revealed a restricted pattern of neuronal differentiation defects at discrete subsets of anatomical scales, including a novel role of cadherins in experience-dependent electrical synapse formation. In total, our analysis results in previously little explored perspectives on cadherin deployment and function.

## Teaser

A comprehensive analysis of cadherin genes in *C. elegans* reveals neuronal differentiation defects at different anatomical scales.

## INTRODUCTION

The emergence of multicellularity was characterized by cell-type diversification and accompanying elaboration of cell adhesion mechanisms (*1*, *2*). Among the most prominent families of cell adhesion molecules (CAMs) are the cadherins, large transmembrane proteins characterized by the presence of multiple protein-protein interaction domains, called cadherin repeats (*3*–*8*). On the basis of primary sequence and/or the presence of additional domains, the cadherin superfamily encompasses multiple subfamilies—namely, classical cadherin, protocadherin, calsyntenin, flamingo, dachsous, fat and fat-like, and cadherin-related (CDHR)—most of which are largely conserved across phyla (*9*–*12*). Cadherin family members orchestrate selective cell-cell recognition and communication events during developmental processes in many tissue types including the brain, ranging from cell migration, axon fasciculation and pathfinding, and synaptic connectivity (*13*–*17*). However, previous expression and functional studies on cadherins in animal brain development were restricted to a subset of cadherins, brain regions, neuronal classes, or developmental stages (*18*–*26*).

The genomic and cellular compactness of *Caenorhabditis elegans* as well as its tremendous genetic toolkit make *C. elegans* an attractive choice to undertake expression and functional analyses of entire gene families, such as the cadherin family. Such comprehensive, gene family- and organism-wide analyses have the potential to reveal common themes in the deployment of specific gene families. For example, the expression and functional analysis of all members of the homeobox gene family revealed their reiterative, combinatorial deployment in neuronal cell-type specification throughout the entire nervous system of *C. elegans* (*27*). Similarly, genome- and animal-wide analysis of neuropeptides and receptors has revealed intriguing perspectives on the abundance of neuropeptidergic communication in the nervous system (*28*) and the nervous system–wide expression analysis of classic neurotransmitter receptor families indicated the extensive and widespread use of nonsynaptic neurotransmitter signaling in the nervous system (*29*). Family-wide analyses of cell adhesion and recognition molecules offer the opportunity to tackle the fascinating problem of how neurons interact with one another to assemble into complex neuronal networks. Such network formation relies on serial cellular interactions on different organizational and anatomical levels, ranging from cell migration and positioning to neurite outgrowth, selective fasciculation, and synapse formation. One particularly intriguing question is whether evolution has selected individual gene families to be dedicated to any or all of these processes. What makes the cadherin family a particularly attractive candidate for a pervasively used determinant of brain patterning is the observation that, unlike for many other gene families involved in other critical neuronal processes (transcription factors, neuropeptides, and ion channels), the number of cadherin genes scales with organismal complexity, suggesting a causal link between the two (*12*).

**Fig. 1.**
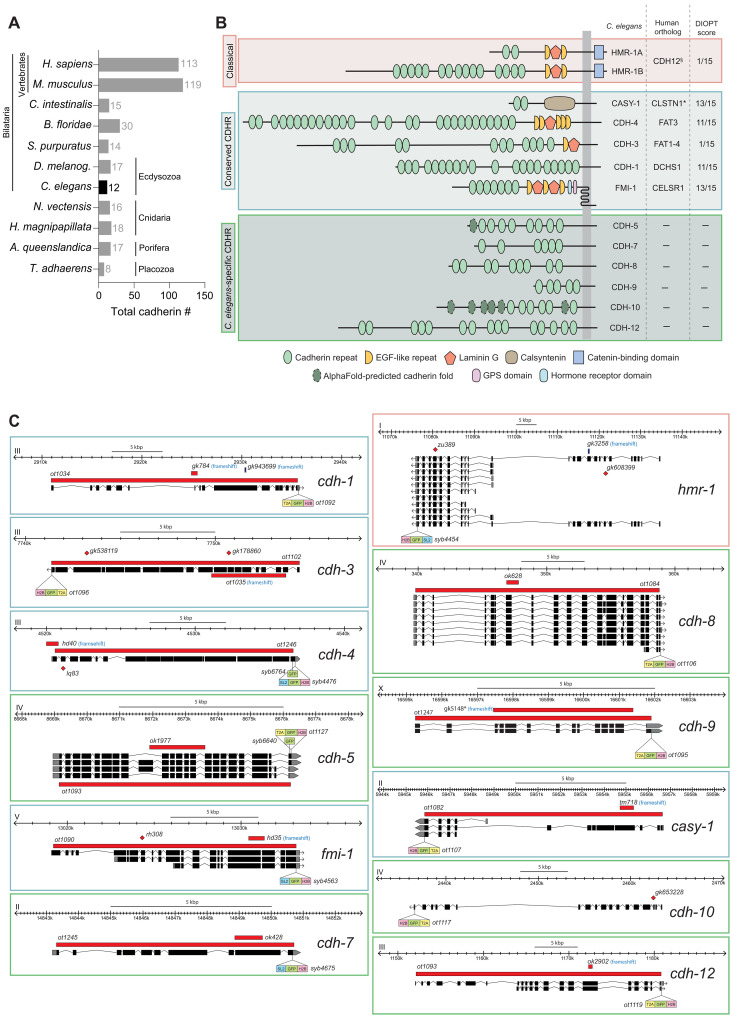
Overview of the cadherin superfamily in *C. elegans*. (**A**) Phylogenetic tree showing the number of cadherin-encoding genes expressed in marked species. *C. elegans* has 12 cadherin genes. (**B**) Schematic representation of *C. elegans* cadherin proteins. The *C. elegans* genome encodes 2 classical and 11 cadherin-related proteins, composed of cadherin repeats (light green ovals) and, in some cases, additional domains (legend). Closest vertebrate orthologs and DIOPT scores (*118*) are listed. Domain structure was retrieved from InterProScan (*119*); additional cadherin folds predicted using AlphaFold (*120*) are shown (dashed dark green ovals). (**C**) Schematic representation of cadherin gene loci and CRISPR-Cas9–engineered reporter and mutant alleles. Each locus was endogenously tagged at the C terminus with a T2A- or SL2-based GFP::H2B cassette to generate nuclear-localized reporters. The viral 2A self-cleaving peptide system uses translational ribosomal skipping to generate separate peptides encoding the endogenous cadherin protein and GFP fused to H2B, whereas SL2 uses trans-splicing to produce two similar transcripts that are independently translated. In some cases, translational reporters were generated by tagging with GFP alone. Null alleles are depicted in red rectangles. Previous alleles, not definitively null, are also annotated.

The *C. elegans* genome encodes 12 cadherin genes (*30*), while mammals encode more than 100 members of the cadherin superfamily, a notable exemplification of how cadherin gene number scales with animal complexity (*12*) (Fig. 1A). While a small subset of the *C. elegans* cadherins has been previously analyzed (*15*, *31*–*44*), the expression and function of several conserved cadherins (for example, the *C. elegans* homolog of Dachsous/DCHS) or the many nonconserved cadherins have not been previously examined at all. To explore how broadly cadherins are used during the construction of the entire *C. elegans* nervous system, we first tagged all 12 *C. elegans* cadherins with a fluorescent reporter protein, using CRISPR-Cas9 genome engineering, and mapped their nervous system–wide expression in the developing and mature nervous system. We find that a subset of cadherins is expressed in all neuron types, while others are selectively expressed in a small subset of neurons. These patterns argue against a widespread use of combinatorial cadherin codes and rather suggest that cadherins fulfill either very broad generic themes in nervous system function (pan-neuronally expressed cadherins) or have very restricted, cell type–specific functions (narrowly expressed cadherins). To explore these possibilities further, we generated null alleles for all cadherins and extensively characterized them for defects in morphology, behavior, neuronal soma positioning, axonal neighborhood topology and fasciculation, axodendritic morphologies, and synapse localization to systematically decode overarching contributions of all cadherin members in establishing and maintaining neuronal structure. Our analysis reveals that different cadherin members play unique roles at various anatomical scales arising from several developmental events.

## RESULTS

### A pan-cadherin nervous system–wide expression atlas

The *C. elegans* genome codes for 12 members of the cadherin family, each characterized by the presence of multiple cadherin repeats (Fig. 1B). Six of the cadherins are conserved across the animal kingdom and include the sole classic cadherin, HMR-1, the Fat orthologs CDH-3 and CDH-4, the Dachsous/DCHS ortholog CDH-1, the CELSR/Flamingo ortholog FMI-1, and the Calsyntenin/Clstn ortholog CASY-1, while another six appear to be nematode specific, in line with frequent species-specific expansion of cadherin family members in other organisms (*9*–*11*). The protocadherin subfamily, characterized by the production of a vast array of isoforms (*45*), is absent in the genome of *C. elegans*, and there is only a very limited number of splice isoforms of *C. elegans* cadherins in general. While some of the conserved *C. elegans* cadherin genes have been characterized for expression and/or function in restricted parts of the nervous system (*15*, *31*–*44*), none of these conserved cadherins have been comprehensively analyzed throughout the nervous system. In addition, none of the six nematode-specific cadherin proteins have been previously analyzed at all in terms of their nervous system expression or function.

**Fig. 2.**
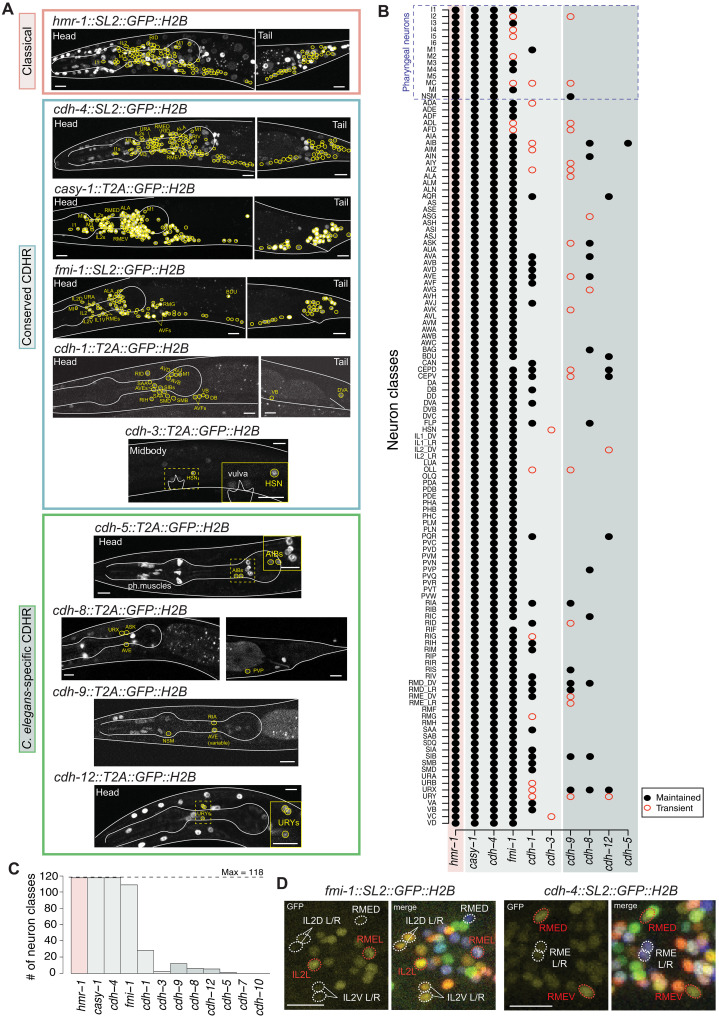
Cell-specific neuronal expression atlas of all cadherins. (**A**) Representative images of cadherin expression in the L4 nervous system. Reporters of classical cadherin *hmr-1*(*syb4454*) (pink) and other cadherins (green) *casy-1*(*ot1108*), *cdh-4*(*syb4476*), and *fmi-1*(*syb4563*) are pan-neuronally or broadly expressed. Reporters of cadherin-related members *cdh-1*(*ot1092*), *cdh-3*(*ot1096*), *cdh-5*(*ot1127*), *cdh-8*(*ot1106*), *cdh-9*(*ot1095*), and *cdh-12*(*ot1119*) are relatively narrowly expressed. All GFP^+^ neurons are encircled in yellow, but only some are labeled. (**B**) Nervous system–wide expression atlas in the L1 and L4 nervous system. GFP^+^ neurons were scored based on overlay with the NeuroPAL landmark reporter (*29*) (*cdh-1* ID example in fig. S1A). Binary presence (black dot) or absence (no dot) of expression was scored. Transient expression is represented in a hollow red circle. (**C**) Abundance of each cadherin in the nervous system. (**D**) Subclass-specific differences in cadherin expression. *fmi-1*(*syb4563*) is enriched in IL2 L/R and RME L/R relative to IL2 D/V and RME D/V, respectively. In contrast, *cdh-4*(*syb4476*) is enriched in RME D/V relative to RME L/R. All images are of L4-stage animals and represent maximum intensity projections of a subset of the Z-stack. A total of 5 to 10 animals were scored for each reporter. Worm body and the pharynx are outlined in white for visualization. Scale bar, 10 μm.

We used CRISPR-Cas9 genome engineering to generate endogenously tagged green fluorescent protein (GFP) reporter alleles for all 12 *C. elegans* cadherin loci (Figs. 1C and 2A). In each case, an SL2::GFP::H2B or T2A::GFP::H2B cassette was inserted at the C terminus to capture expression of all putative splice isoforms, which all share common 3′ exons (Fig. 1C). Because we expect many cadherins to display restricted subcellular expression patterns, including at synapses and neurites, we fused histone 2B (H2B) to GFP to localize reporter signals to the nucleus, allowing us to unambiguously map the identity of cellular sites of expression using a polychromatic landmark reporter transgene, NeuroPAL (Neuronal Polychromatic Atlas of Landmarks) (*29*) (Fig. 2A and fig. S1A).

We first mapped expression in the nervous system at late larval and young adult stages, when most of the nervous system has fully matured (Fig. 2B and table S1). Overall, we found that cadherin expression patterns fall into two distinct categories: broad versus narrowly expressing (Fig. 2, A to C). Of the 12 cadherin genes, 4 were found to be expressed either pan-neuronally (*hmr-1*, *cdh-4/Fat*, *casy-1/Clstn*) or very broadly but not quite pan-neuronally (*fmi-1/Celsr*). Notably, all neuron types in which the *Celsr*/Flamingo homolog *fmi-1* is not expressed are located in the enteric (pharyngeal) nervous system of *C. elegans*, which has been proposed to constitute a more primitive domain of animal nervous systems (*46*, *47*).

The remaining eight cadherins are narrowly expressed, but at varying levels of sparsity: four cadherins (the conserved *cdh-1/Dchs* and the nematode-specific *cdh-8*, *cdh-9*, and *cdh-12*) are expressed in 5 to 27 neuron classes (out of a total of 118 in the nervous system of the hermaphrodite), two are expressed in only 1 to 2 neuron classes (*cdh-3/Fat* in HSN and VC, and *cdh-5* in AIB), while the remaining two (*cdh-10* and *cdh-7*) do not show any detectable neuronal expression (Fig. 2, A to C, and fig. S1B). There is no correlation of neuron-specific cadherin expression with genetic relatedness of neurons, as assessed by neuronal clustering based on scRNA-seq expression (fig. S1C) (*48*). In addition, cadherin expression is not enriched in specific parts of the nervous system (sensory periphery, interneuron, and motor circuit).

Expression levels of individual cadherins show stable differences across neuronal classes for each individual cadherin gene and reflect quantitative differences reflected in scRNA-sequencing data (Fig. 2A and fig. S1D) (*48*). Expression differences can also be observed within members of the same neuronal class. For example, sensory neuron class IL2 and motor neuron class RME—both of which are further divided into subtypes based on anatomy and function—show differential cadherin expression across subtypes (Fig. 2D) (*49*–*52*). The cadherin *fmi-1*/*Celsr* is enriched in IL2 L/R and RME L/R relative to IL2 D/V and RME D/V, respectively, and cadherin *cdh-4*/*Fat* is enriched in RME D/V relative to RME L/R. The in vivo relevance of quantitative expression differences in genes encoding CAMs—specifically cadherins—has been previously reported, such as in the case of Flamingo in *Drosophila melanogaster* (*26*).

Altogether, 61 of the 118 neuron classes of the late larval/adult nervous system do not express cell type–specific cadherins beyond the four broadly expressed cadherins *hmr-1*, *cdh-4*, *casy-1*, and *fmi-1*. To frame this finding in the context of our original question of the expression specificity of the *C. elegans–*specific cadherins, we can conclude that cadherins do not form pervasive neuron type–specific codes of coexpression, at least in the mature nervous system.

**Fig. 3.**
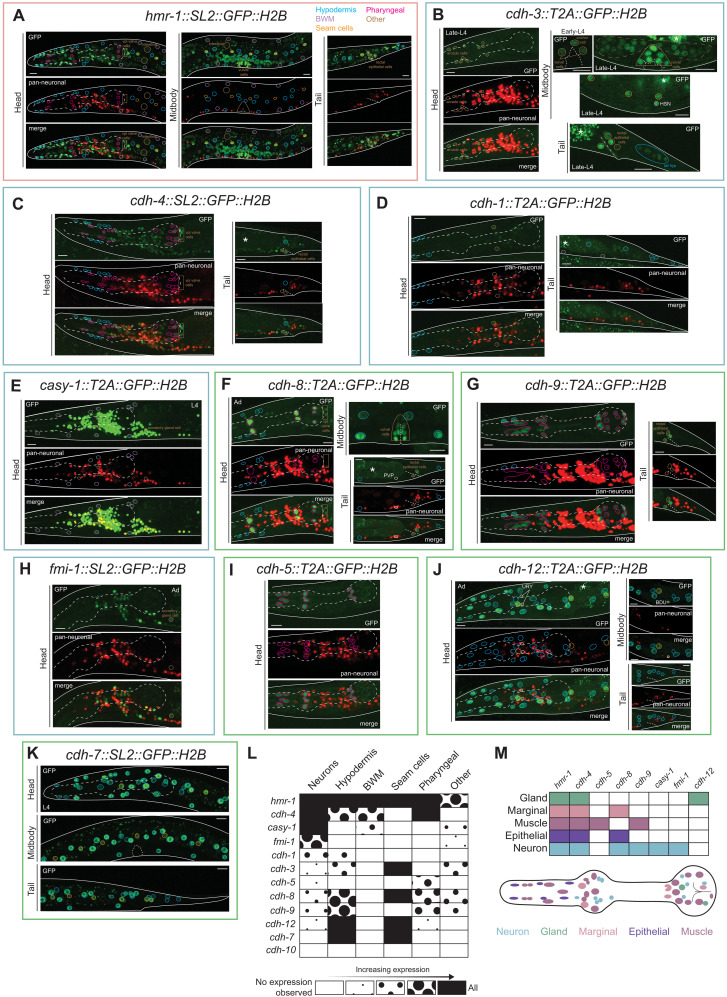
Cadherins are differentially expressed in nonneuronal tissues. Expression of cadherins in nonneuronal tissues, including the hypodermis (blue), muscles (purple), pharyngeal nonneuronal (pink), seam cells (orange), and others (brown). (**A**) *hmr-1* is expressed broadly expressed in all nonneuronal tissues. (**B**) *cdh-3* is expressed in vulval muscles, anchor cell, utse cell, seam cells, rectal epithelial cells, hypodermal cells in the tail, and arcade cells. (**C**) *cdh-4* is broadly expressed in multiple nonneuronal tissues, including hypodermal, pharyngeal, and rectal epithelial cells. (**D**) *cdh-1* is expressed in the hypodermis and seam cells. (**E**) *casy-1* is expressed in body wall muscles, although expression is significantly dimmer compared to neuronal expression. (**F**) *cdh-8* is expressed in pharyngeal, vulval, hypodermal cells, and the pharyngeal-intestinal valve (vpi). (**G**) *cdh-9* is expressed in various pharyngeal cells. (**H**) *fmi-1* is expressed in the excretory gland cell. (**I**) *cdh-5* is expressed in pharyngeal muscle cells. (**J** and **K**) *cdh-12* (J) and *cdh-7* (K) are expressed in hypodermal cells throughout the length of the worm. *cdh-7* is the only cadherin that is expressed exclusively in nonneuronal cells. (**L**) Summary of the extent of cadherin expression in various tissues, broadly categorized. Glial cells are excluded because glia-specific reporters have not yet been analyzed. (**M**) Cadherins are combinatorially expressed in nonneuronal cells of the pharynx. All images are of L4/Ad-stage animals and represent maximum intensity projections of a subset of the Z-stack. A total of 5 to 10 animals were scored for each reporter. Gut autofluorescence is marked with an asterisk. Unidentified cells are circled in white. Scale bar, 10 μm.

There is no cadherin whose expression is restricted exclusively to the nervous system (summarized in Fig. 3L). Outside the nervous system, *hmr-1* is the most broadly expressed cadherin, although not every cell expresses it (Fig. 3A). Differential cadherin expression profiles in nonneuronal tissues include the hypodermis (the *C. elegans* epidermis), muscles, nonneuronal cells of the pharynx, seam cells, epithelial cells, and arcade cells (Fig. 3, A to K). Epidermal cells are clearly the most predominant site of nonneuronal cadherin expression, with *cdh-7* and *cdh-12* showing the greatest similarities in their broad epidermal expression (Fig. 3, J and K). Within the same type of nonneuronal tissue, cadherins are occasionally expressed in a subset of constituting cell types. The pharynx, the foregut of the worm that mediates food intake, is composed of a highly diverse set of cells, including 14 neuron classes and muscle, epithelial, gland, and marginal cells; in addition to neuronal expression (see above), we analyzed cadherin expression in all nonneuronal pharyngeal cell types and found notable patterns of cellular specificity in cadherin expression profiles (Fig. 3M).

### Spatiotemporal changes in cadherin expression

Temporal changes in cadherin expression have been previously reported in many contexts (*53*–*56*). However, these studies were restricted to a small subset of cadherins, mostly classical cadherins, and were not performed with sufficient cellular specificity, on a nervous system–wide level, or across postnatal development. None of the *C. elegans* cadherins have been comprehensively assessed for expression dynamics with single neuron resolution. To fill these gaps, we sought to systematically assess changes in neuronal cadherin expression between developing and mature stages, with a focus on the developing nervous system.

**Fig. 4.**
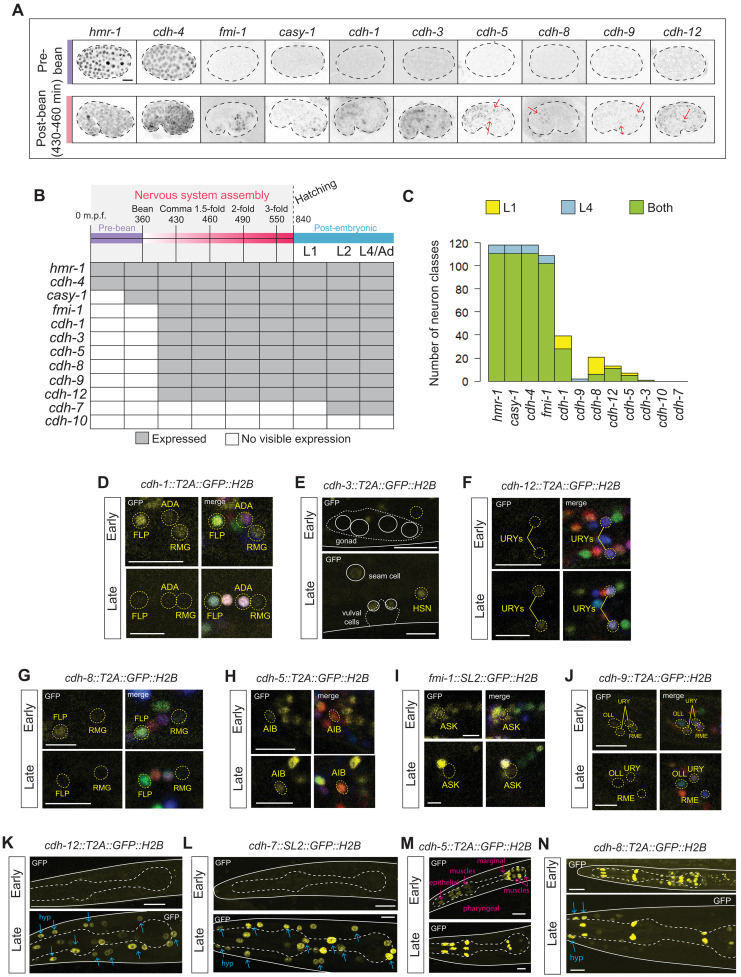
Widespread temporal changes in cadherin expression across development. (**A**) Differential onset of cadherin expression. All nonclassical cadherins, besides *cdh-4*(*syb4476*), show detectable expression at the bean (*casy-1*) or post-bean (remaining cadherins shown) embryonic stage, and continue expressing at posthatch (L1 stage); notably, onset of expression of cadherin-related genes coincides with neuronal differentiation and nervous system assembly. *cdh-7* is not expressed at any embryonic stage but starts expressing in nonneuronal tissues at the L2 larval stage (fig. S1B). No visible expression was observed for *cdh-10* at any stage in development (fig. S1B), corroborating scRNA-seq data (*48*, *61*). (**B**) Embryonic expression of all cadherin reporters in early and mid/late embryogenesis. (**C**) Comparative abundance of each cadherin in the nervous system of L1- and L4-stage animals. L1-only, L4-only, and shared expression are denoted in yellow, blue, and green, respectively. (**D** to **J**) Representative examples of temporal transitions in cadherin expression in the nervous system. Cadherins *cdh-1*(*ot1092*), *cdh-8*(*ot1106*), *fmi-1*(*syb4563*), and *cdh-9*(*ot1095*) are down-regulated between early and late stages of development. Conversely, cadherins *cdh-3*(*ot1096*), *cdh-12*(*ot1119*), and *cdh-5*(*ot1127*) are up-regulated. Expression changes are neuron specific. (**K** to **N**) Representative examples of temporal transitions in cadherin expression in nonneuronal tissues. Cadherins *cdh-7*(*syb4675*) and *cdh-12*(*ot1119*) begin expressing in the hypodermis (blue) at the L2 stage. *cdh-5* shows restricted and enriched expression in the pharynx (pink) at late stage. *cdh-8* is up-regulated in the hypodermis at late stage. All images are maximum intensity projections of a subset of the Z-stack. Gut autofluorescence is marked with an asterisk. Scale bar, 10 μm.

We found that of the 12 cadherins, only 2—the conserved classic cadherin *hmr-1* and the conserved *cdh-4/Fat* cadherin—are expressed early in embryogenesis, in dividing cells (Fig. 4, A and B); during this proliferative, premorphogenetic stage, many short-range cell migratory events occur throughout the embryo, termed “cell focusing” (*57*, *58*), as well as gastrulation. Early embryonic expression of these two cadherin genes correlates with *hmr-1/classical cadherin* and *cdh-4/Fat* mutants being the only cadherin mutants with prominent embryonic lethality, as we describe further below (*37*, *59*). The onset of all other cadherins is observed at about the time when most cells have started to exit the cell cycle and overall nervous system morphogenesis commences (Fig. 4, A and B).

Because the contorted shape and vigorous movement of late-stage embryos in the eggshell severely limit the ability to precisely identify reporter gene expression patterns with reliable single-cell resolution, we inferred cadherin expression at these critical stages by making use of the documented multihour (~6 hours) stability of the H2B moiety in our reporter cassette (*60*). Such stability will lead a GFP::H2B reporter that turned on expression postmitotically at mid/late embryogenesis to perdure until the initial hours of the first larval stage (L1). We therefore identified cadherin expression patterns at the L1 stage, with single neuron resolution using the NeuroPAL reporter, to infer cadherin expression at mid/late-embryonic stages when neuronal development and circuit formation occur (Fig. 2B, fig. S2A, and table S1). The notion that expression patterns of H2B-based reporters at the first larval stage accurately reflect expression at earlier, mid-embryonic stages is further supported by two observations: (i) timing of cadherin expression onset visualized by our CRISPR reporters supports a previous embryonic single-cell RNA sequencing (scRNA-seq) dataset, and (ii) our pan-cadherin expression atlas at the first larval stage and the embryonic scRNA-seq data show a similar dichotomy of broadly versus narrowly expressed cadherins, with largely congruent sites of expression; in most cases where we saw discrepancy, expression had typically already decreased by late embryogenesis and was, hence, not captured by L1 stage (table S2) (*61*).

Our L1 stage cadherin expression atlas leads us to conclude that cadherin expression is, to some extent, more widespread at developing stages compared to the L4/Ad nervous system. Specifically, we found that 10 cadherins are expressed in the developing nervous system, 5 of which (*fmi-1/Celsr*, *cdh-1/Dchs*, *cdh-8*, *cdh-9*, and *cdh-12*) are more broadly expressed at the developing stage (Fig. 4C and fig. S2A). Of the remaining cadherins, three (*hmr-1*, *cdh-4/Fat*, and *casy-1/Clstn*) did not show significant changes in expression between developing and mature stages (Fig. 4C). We did not find any detectable expression for cadherins *cdh-7* and *cdh-10* at the first larval stage (fig. S1B). While there is generally broader expression of cadherin expression during embryonic development of the nervous system, we consider it notable that 45 out of all embryonically generated neuron classes fail to express any cadherin other than the broadly expressed cadherins *hmr-1*, *cdh-4/Fat*, *casy-1/Clstn*, and *fmi-1/Celsr*. As stated above already in the context of the adult nervous system, we can conclude that cadherins do not form pervasive neuron type–specific codes of coexpression.

We found that changes in cadherin expression and directionality of change were gene and neuron class specific (Fig. 4, D to J). For instance, FLP sensory neurons express *cdh-1/Dchs* and *cdh-8* very highly at the developing stage but minimal or no expression, respectively, was detected at the late larval/early adult stages (Fig. 4, D and G). Similarly, *fmi-1/Celsr* is expressed more broadly at the developing stage compared to the mature stage (Fig. 4I). While expression of most cadherins decreased between L1 and L4/adult stages, we observed an increase in expression in some cases. For example, *cdh-12* expression in URY sensory neurons increased across development (Fig. 4F). *cdh-5* expression in AIB interneurons also increased across development but decreased in the late larval-to-adult transition, a window in which we found relatively few changes in cadherin expression (Fig. 4H and fig. S2B). Of note, when dynamic, expression did not necessarily change progressively in one direction. In the case of *cdh-5* in AIB neurons, for example, expression increased from L1 to L4, and then decreased in adults (fig. S2B).

Last, we observed additional changes in cadherin expression outside the nervous system (Fig. 4, K to N). The most notable change was in *cdh-7* and *cdh-12* expression; while no expression was observed at the L1 stage, both genes turned on their hypodermal expression at the start of larval stage L2, after many cells of the lateral hypodermis have stopped dividing (fig. S2C).

### Mechanisms of spatiotemporal regulation of cadherin expression

Many cadherins are expressed in a neuron type–specific manner and thus contribute to transcriptomic diversity in the nervous system (Figs. 2 and 4). Extensive studies on the transcriptional regulation of neuron-specific terminal gene batteries have shown that terminal selector–type transcription factors are key regulators of postmitotic neuronal identities (*62*). While regulation of many gene families—including genes encoding CAMs such as immunoglobulin, neurotransmitter pathway, and other synaptic machinery molecules—has been pursued, cadherins are sparingly represented in these studies owing to a lack of reliable reporter reagents and the limited knowledge of expression patterns before this work (*49*, *63*, *64*).

**Fig. 5.**
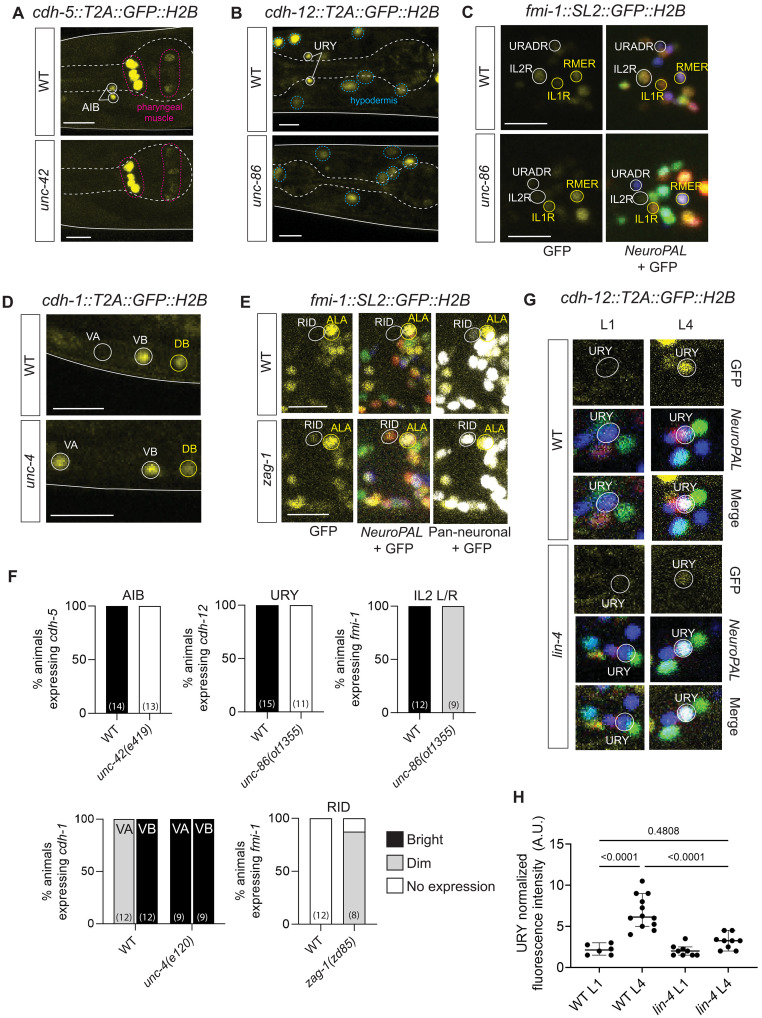
Spatiotemporal regulation of cadherin expression in the nervous system. (**A** to **E**) Representative images of cadherin reporters in wild-type and mutant backgrounds. *cdh-5::T2A::GFP::H2B* (*ot1127*, AIB), *cdh-12::T2A::GFP::H2B* (*ot1119*, URY), and *fmi-1::SL2::GFP::H2B* (*syb4563*, only IL2R scored) are down-regulated in mutants of terminal selector transcription factors UNC-42 (allele *e419*) and UNC-86 (*ot1355* for *cdh-12*, *ot1354* for *fmi-1*). *cdh-1::T2A::GFP::H2B* (*ot1092*) and *fmi-1(syb4563)* expression is derepressed in neurons VA and RID in transcription factors mutants *unc-4(e120)* and *zag-1(zd85)*, respectively. *cdh-1–* and *fmi-1–*expressing neurons were identified with NeuroPAL overlay. (**F**) Quantification of cadherin expression for (A) to (E). Statistical significance was assessed using the Fisher’s exact test or chi-squared test. (**G** and **H**) Representative images (G) and quantification (H) of *cdh-12*(*ot1119*) in wild-type and *lin-4*(*e912*) mutant backgrounds in URY neurons. In wild-type animals, *cdh-12* expression increases between L1 and late L4 stages. *cdh-12* expression in *lin-4* mutant L4 animals is significantly reduced and resembles L1 expression levels. All images are maximum intensity projections of a subset of the Z-stack. Scale bars, 10 μm.

We spot checked whether cadherin expression is controlled by terminal selector–type transcription factors and indeed found this to be the case in several distinct cellular contexts. The terminal selector of AIB neuron identity, *unc-42* (*65*), controls *cdh-5* expression in AIB interneurons (Fig. 5, A and F)*. cdh-12* expression in the URY sensory neurons is lost in animals lacking the terminal selector of URY identity, the *unc-86* POU homeobox gene (*66*), and similarly, expression of *fmi-1/Celsr* in IL2 sensory neurons, where *unc-86* also acts as a terminal selector (*67*, *68*), is affected in *unc-86* mutants (Fig. 5, B, C, and F).

Motor neuron diversity in the ventral nerve cord of *C. elegans* is controlled by sets of repressor proteins that antagonize the function of the pan-cholinergic motor neuron selector *unc-3/EBF* (*69*). We found that cadherins are controlled by a similar transcriptional regulatory logic. *cdh-1*—typically expressed in both lineally related sister neurons VA and VB but enriched in VB neurons—was derepressed in VA neurons in animals lacking the homeobox repressor *unc-4* (*70*), a known subtype selector that distinguishes VA from VB neurons (Fig. 5, D and F). Another broadly used repressor of neuronal identity features, the *zag-1* Zn finger/homeobox gene (*71*, *72*), also controls cadherin expression as we find that *fmi-1/Celsr* expression is derepressed in RID neurons in *zag-1* mutants (Fig. 5, E and F). We conclude that cadherins are (i) substrates of a diverse set of transcriptional regulatory routines previously shown to generate spatial diversity of neuronal gene expression programs, and (ii) markers of fully differentiated neuron types and, like other cell-specific neuronal genes, are tightly coupled with neuronal identity.

On the temporal axis, pathways that regulate changes in CAM gene expression are not well understood. Recent transcriptomic studies reveal the significant contribution of heterochronic pathway genes and ecdysone hormone signaling in mediating temporal expression changes in the postmitotic nervous system of *C. elegans* and *D. melanogaster*, respectively (*73*, *74*). We asked whether cell-specific temporal changes in cadherin expression were regulated by the heterochronic pathway. The heterochronic pathway comprises a cascade of miRNAs and proteins, including the miRNA *lin-4* that is required to down-regulate the transcription factor LIN-14 to promote the temporal transition between early larval stages L1 and L2 (*75*). To assess whether temporal changes in cadherin expression are regulated by this pathway, we focused on *cdh-12*. In wild-type animals, *cdh-12* expression in URY sensory neurons increases significantly between L1 and L4 developmental stages (Figs. 4F and 5, G and H). *lin-4* up-regulation in the L1-to-L2 transition period is required to suppress juvenile and promote late-stage neuronal identity features, and we indeed find that *cdh-12* expression is maintained in its juvenile “off stage” in *lin-4* mutants (Fig. 5, G and H). Thus, the expression of *cdh-12* in URY neurons is regulated by intersecting roles of the transcription factor UNC-86 and the heterochronic pathway on spatial and temporal axes, respectively.

### Mutant analysis of *C. elegans* cadherins

We used CRISPR-Cas9 genome engineering to generate mutant alleles of each individual cadherin locus (Fig. 1C), except for the broadly and early expressed classical cadherin *hmr-1*, a previously well-characterized essential gene required for global embryonic morphogenesis (*31*, *32*, *41*) and the nematode-specific cadherins *cdh-7* and *cdh-10*, for which we detected no neuronal expression at any stage. For several of these nine cadherins, no mutant allele has either existed or been examined before and for several others—including the conserved cadherins—previously used mutant alleles (mostly small deletions) are not unambiguous molecular null alleles. Hence, our strategy consisted of deleting the entire locus for each of the nine cadherin genes.

We found that of all the nine null mutant cadherin strains, only *cdh-4/Fat* null mutants display embryonic and larval lethality, albeit in an incompletely penetrant manner. As described with previous alleles, these embryonic and larval arrest phenotypes appear to be a consequence of severe morphogenetic defects (*37*). Hence, the only two essential cadherins, *cdh-4/Fat* and *hmr-1*, that are required for embryonic development are those that are expressed throughout early and late embryonic development.

All other cadherin null alleles are viable, fertile, and appear overall healthy. With the exception of *cdh-3/Fat* null mutants, which display tail morphogenetic defects as described previously (*76*) and *cdh-4/Fat* null animals that escape embryonic/larval arrest, no other cadherin mutants displayed obvious morphological defects. Again, except for *cdh-4/Fat* escapers, none of the cadherins display the types of obvious uncoordinated (Unc) locomotor defects that are characteristic for mutants of many genes involved in nervous system development or function. We subjected all mutants to a more detailed quantitative locomotory analysis using a MultiWorm tracker system that measures multiple locomotory parameters (*77*). We observed defects in a subset of locomotory behaviors in *fmi-1/Celsr*, *cdh-4/Fat*, and *cdh-5* null mutants but observed limited/no defects in animals lacking the pan-neuronally expressed, conserved *casy-1/Clstn*, *cdh-1/Dchs,* or the nematode-specific *cdh-8 and cdh-9* cadherins (table S3).

In the ensuing sections, we describe a more detailed mutant analysis of the two conserved, broadly neuronally expressed *cdh-4/Fat* and *fmi-1/Celsr* and of the more cell-type specifically expressed conserved *cdh-1/Dchs* and *cdh-3/Fat*, as well as of the nematode-specific *cdh-5*, *cdh-8*, *cdh-9*, *and cdh-12* genes. Several aspects of *cdh-4/Fat* and *fmi-1/Celsr* had already been previously described and we broaden this analysis here further, while the other six cadherins had not been examined for nervous system development before. We excluded from this more detailed analysis the two pan-neuronally expressed cadherins whose function in nervous system development and function have been extensively described before, namely, *hmr-1* (*31*, *32*, *41*) and *casy-1/Clstn* (*38*–*40*, *42*–*44*), as well as the nonneuronally expressed, nematode-specific *cdh-7.*

### The conserved cadherin *cdh-4/Fat* affects neuronal morphogenesis at multiple organizational levels

**Fig. 6.**
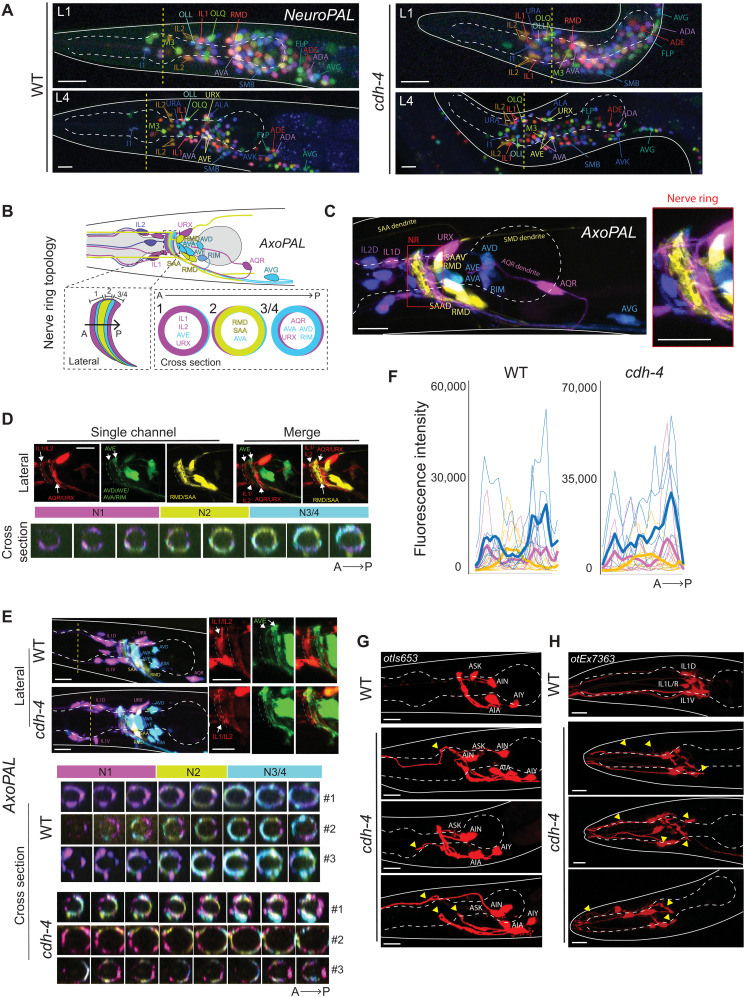
Phenotypic characterization of *cdh-4/Fat* mutants. (**A**) Neuronal soma mispositioning defects in *cdh-4(ot1246)* mutants. Representative images of soma positions in the landmark reporter NeuroPAL (*otIs669*) are shown. For reference, the middle of the anterior pharyngeal bulb is marked with a yellow dashed line. (**B** and **C**) AxoPAL reporter design for assessing axonal topology in the nerve ring. Schematic illustration (B) and a representative image (C) of AxoPAL reporter (*otEx7895*). A subset of neuron classes is cytoplasmically labeled with four distinct fluorophores to visualize three distinct neighborhoods in the nerve ring: N1 (magenta), N2 (yellow), and N3/4 (cyan) along the anteroposterior axis. (**D**) Lateral and cross-sectional representations of AxoPAL to depict positioning of labeled neurons in distinct axonal neighborhoods. IL1 and IL2 occupy the anterior-most neighborhood, N1. Neurons AVD, AVE, AVA, RIM, AQR, and URX largely occupy N3/4; the neighborhood shift of the command interneuron AVE is visible. Neurons RMD and SAA occupy neighborhood N2, which is distinct from N1 and N3/4. (**E**) *cdh-4(ot1246)* mutants have variable defects in axonal topology. Representative images of lateral and cross-sectional views of AxoPAL are shown. (**F**) Neighborhood-constituent reporter intensity traces along the A-P axis in *cdh-4(ot1246)* and WT animals. Bold line graphs represent an average of multiple worms. (**G** and **H**) Variable axodendritic defects in AIN (G) and IL1 (H) neurons. All images are maximum intensity projections of a subset of the Z-stack. A total of 5 to 10 animals per genotype were analyzed for the NeuroPAL and AxoPAL analyses. Scale bars, 10 μm.

*cdh-4/Fat* null mutants display a partially penetrant embryonic or early larval arrest phenotype characterized by severe morphological malformations (*37*). Surviving animals display several neuronal phenotypes, including axon fasciculation and neuronal migration defects (*36*, *37*). However, these previous analyses were restricted to a few neuron classes. To further expand mutant analyses across neuron types and anatomical structures, we next broadly assessed neuronal architecture—including soma positions, axon neighborhood placement, neurite contact, and synapse structure—in *cdh-4/Fat* mutants (Fig. 6). *cdh-4/Fat* was previously implicated in neuronal migration in a subset of postembryonically born midbody neurons that undergo long-range migrations (*36*, *37*). We thus asked whether the migratory phenotype extended to most neuron classes that undergo short-range migration during embryogenesis. Neuronal soma positions were assessed using the fluorescent transgenic tool NeuroPAL (*29*). Although positions were scored in the L4-stage nervous system for ease of scoring, our analysis considered the overall developmental history of cadherin expression in each neuron studied because we reasoned that a positioning defect induced at the embryonic or early postembryonic stages would persist until maturity. We found that neurons located in the anterior ganglion had mispositioned soma in two independent *cdh-4/Fat* mutant alleles—our full deletion allele *ot1246* and allele *hd40*; quantification of neuronal displacement in the anterior ganglion showed a subset of neurons being anteriorly displaced in *cdh-4* mutants, with the remaining neurons being unchanged relative to one another (Fig. 6A and fig. S3, A and B). Neurons were already mispositioned in L1 animals right after hatching, indicating that the phenotype is developmental rather than a failure to maintain initially correct neuronal positions until late larval/adult stages (Fig. 6, A and B). Neuronal defects in mice with mutations in the *cdh-4* homolog *Fat* arise from overproliferation of progenitors and their failure to differentiate into neurons (*78*), but we did not find similar phenotypes in our NeuroPAL analysis: *cdh-4(ot1246)* mutants and wild-type animals had the same number of neurons in the head, assessed based on a synthetic pan-neuronal reporter included in the NeuroPAL strain (fig. S3C).

Given prior evidence of cell body positions being correlated with layered nerve ring axonal topology (*79*), we reasoned that defects in neuronal soma positioning in *cdh-4/Fat* mutants would, in turn, perturb nerve ring organization. Typically, such analysis would rely on multiple reporters that fluorescently label single or very sparse subsets of neurons; this is tedious and precludes analyzing relative axon positions in process-dense neuropils such as the *C. elegans* nerve ring. Conversely, multicolor reporters that selectively label neuronal subsets not only expedite analysis but provide relative spatial information (*80*). We therefore engineered a multicolor axonal reporter, which we term “AxoPAL,” that cytoplasmically labels a subset of head neurons (IL2, IL1, URX, AVA, AVE, AVD, RIM, AVG, RMD, SAA, and AQR) in four distinct colors to be able to spatially disambiguate their nerve ring axonal placement (Fig. 6, B and C). Briefly, AxoPAL resolves three distinct nerve ring neighborhoods (N1, N2, and N3/4 from left to right along the AP axis) both laterally and cross-sectionally (Fig. 6D). Anterior ganglion inner labial neurons (IL1 and IL2) project axons posteriorly to mainly occupy N1 and loop across all posterior neighborhoods. Neurons in ganglia posterior to the nerve ring project anteriorly; these include RMD and SAA (N2, yellow), and AVA, AVE, AVD, RIM, AQR, and URX (N3/4). AVE, URX, IL1, and IL2 discernibly occupy multiple neighborhoods in AxoPAL, which is consistent with previous electron micrographical studies (Fig. 6D) (*52*, *79*, *81*). Unlike the nuclear reporter NeuroPAL, AxoPAL labels only a subset of neurons to achieve sufficient spatial resolution to visualize neuronal processes. AxoPAL-labeled neurons were chosen based on each class expressing a subset of narrowly expressing cadherins at one or multiple time points in development (Fig. 2B).

In *cdh-4/Fat* mutants, we found that the neuron-specific axonal composition in each neighborhood was disrupted, such that distinctive neighborhoods failed to be established, with the most obvious disorganization defects in interneurons (AVA/AVE/AVD/RIM labeled in cyan); in addition, the AQR axon was missing in most animals because the cell body fails to migrate anteriorly in the absence of *cdh-4/Fat* as shown previously (*37*) (Fig. 6, E and F). Apart from disrupted neighborhood topology, the nerve ring was anteriorly shifted in a subset of *cdh-4/Fat* mutant animals (fig. S3D). On the macroscopic organizational level, we also observed variable defects in axodendritic morphologies—as shown for neuron classes AIN and IL1—in *cdh-4* mutants (Fig. 6, G and H).

### Effects of *cdh-4/Fat* on synaptic structure

Extending our analysis of neuronal anatomy to finer structures, we next asked whether loss of CDH-4/Fat affects synaptic structure. Here, we took advantage of a recently developed repertoire of synaptic reporters comprising two types of reporters: (i) reporters that visualize all presynaptic specializations of a neuron via fluorescently tagged synaptic proteins such as CLA-1/Piccolo or RAB-3 to label all synapses made by that neuron, and (ii) reporters that use split-fluorophore technology, such as GRASP and iBLINC, to specifically label synapses between two neuron types (*82*–*84*).

**Fig. 7.**
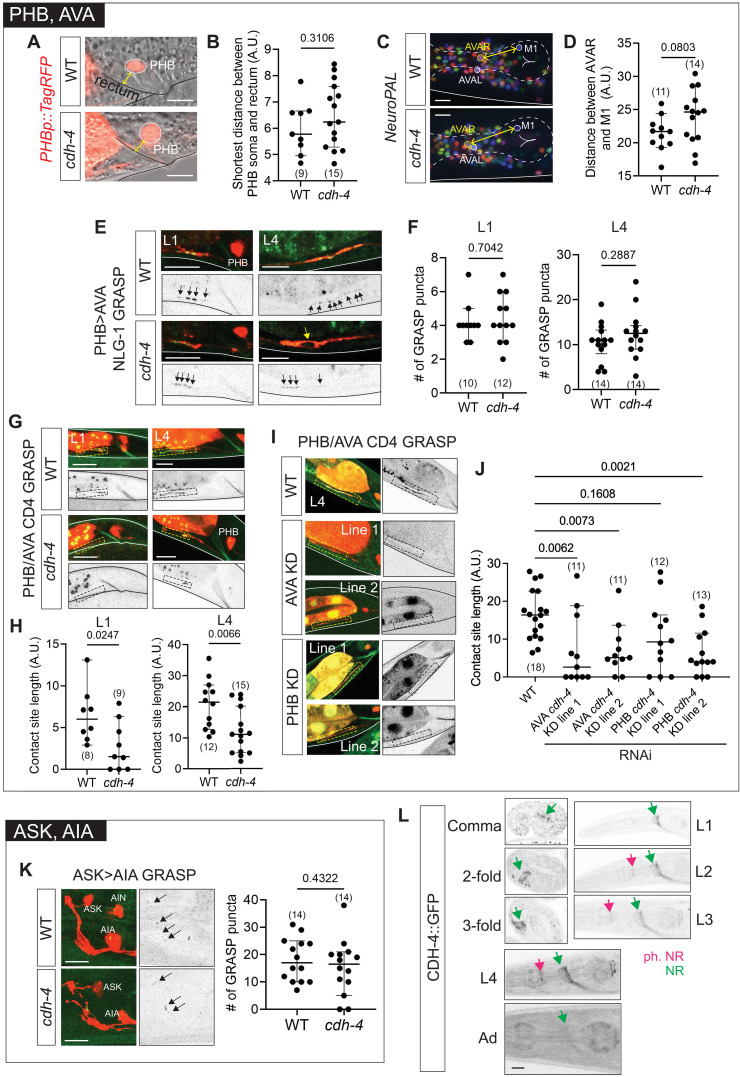
*cdh-4* mutants have context-specific effects on neuronal soma positions, axonal fasciculation, and synapse formation. (**A** and **B**) PHB soma positions are unaffected in *cdh-4* null mutants. Representative images (A) and quantification (B) of PHB soma labeled with *srab-20p::TagRFP* (*otEx8152*) in wild-type and *cdh-4(ot1246)* null mutants. The shortest distance (yellow) between PHB soma and the rectum was quantified in L4-stage animals. (**C** and **D**) AVA soma positions are unaffected *cdh-4* null mutants. Representative images (C) and quantification (D) of AVAR soma labeled with *NeuroPAL* (*otIs669*) in L4-stage wild-type and *cdh-4(ot1246)* null mutant animals. The shortest distance (yellow) between AVAR and the M1 soma, both in the same *Z* plane, was quantified to assess AVA soma position. M1 position is unaffected. (**E** and **F**) PHB>AVA synapses (GRASP strain *otIs839*) are unaffected in *cdh-4* null mutants (L1 and L4 stages). The yellow arrow shows PHB-AVA axonal de-fasciculation and corresponding exclusion of synapses. (**G** and **H**) Contact length (adjacency) between PHB and AVA neurons, measured with PHB-AVA CD4 GRASP reporter *otEx8152*, is reduced in *cdh-4(ot1246)* null mutants (L1 and L4 stages). Dashed box represents PHB and AVA contacts. (**I** and **J**) Contact length between PHB and AVA after cell-specific AVA (*otEx8309* and *otEx8310*) and PHB (*otEx8307* and *otEx8308*) RNAi *cdh-4* knockdown (KD). Two independent lines were tested for each neuron. Dashed box represents the site of PHB and AVA contact. *P* values shown were calculated using one-way ANOVA with Bonferroni correction. (**K**) ASK>AIA synapses are unaffected in *cdh-4(hd40)* mutants. (**L**) CRISPR-tagged CDH-4::GFP shows diffuse expression in the nerve ring. All images are maximum intensity projections of a subset of the Z-stack. Scale bars, 10 μm. In all graphs, a dot represents one worm and error bars denote median with 95% confidence interval. *P* values from unpaired *t* test are shown in (B), (D), (F), (H), and (K).

First, we studied the role of *cdh-4/Fat* in the context of PHB and AVA synapses. We analyzed multiple features of PHB and AVA development to assess whether they are dissociable. *cdh-4/Fat* null mutants did not show a significant PHB/AVA soma positioning defect (Fig. 7, A to D) and the number of synapses between PHB and AVA—assessed with an NLG-1 GRASP reporter—was also unaffected at both L1 and L4 stages (Fig. 7, E and F). We observed that PHB>AVA synapses in *cdh-4/Fat* mutants were clustered in regions of PHB/AVA fasciculation and excluded where PHB and AVA axons lost contact (Fig. 7E). This prompted us to ask whether CDH-4/Fat’s role in mediating PHB/AVA neurite contact and synapse formation could be dissociated. We found that PHB-AVA neurite contact—visualized with a CD4-GRASP reporter that we previously engineered (*85*)—was significantly reduced in *cdh-4/Fat* mutants; again, this phenotype was present at both L1 and L4 stages, suggesting that it is not a defect in the maintenance of neurite contact, rather the establishment of contact is affected early in development (Fig. 7, G and H). To test whether the role of *cdh-4* in mediating PHB-AVA contact is cell-autonomous or nonautonomous, we performed cell-specific RNAi knockdown of *cdh-4* in PHB and AVA (Fig. 7, I and J). Our results suggest that CDH-4/Fat acts in both AVA and PHB to mediate contact between PHB and AVA but is dispensable for synapse formation.

To broaden our analysis to other neuron types, we assessed the role of *cdh-4/Fat* in forming synapses between additional neuron pairs. Synapses were unaffected in an ASK>AIA GRASP reporter in *cdh-4/Fat* mutants (Fig. 7K). *cdh-4/Fat* null mutants have mispositioned IL2/IL1 somas—assessed by scoring the distance between IL2VR and IL1VR—with the distance between the two neuron somas being greater in mutants (fig. S3, E and F). Despite the mispositioned somas, however, IL2>IL1 synapses—visualized with an NLG-1 GRASP reporter—could still be observed (fig. S3G).

Together with the lack of phenotype in PHB>AVA synapses, these results implicate CDH-4/Fat in mediating cell-specific neuronal soma positions and/or neurite contact, but not synapse specificity or synapse formation, at least in the specific contexts that we tested here. A role in determining neurite contact is also supported by CDH-4 localization patterns. A CRISPR-engineered CDH-4::GFP reporter shows visibly diffuse GFP expression along membranes in the nerve ring bundle at all stages of development (Fig. 7L).

### The conserved cadherin *fmi-1/Celsr* controls axodendritic morphologies, axon pathfinding, and synapse formation

**Fig. 8.**
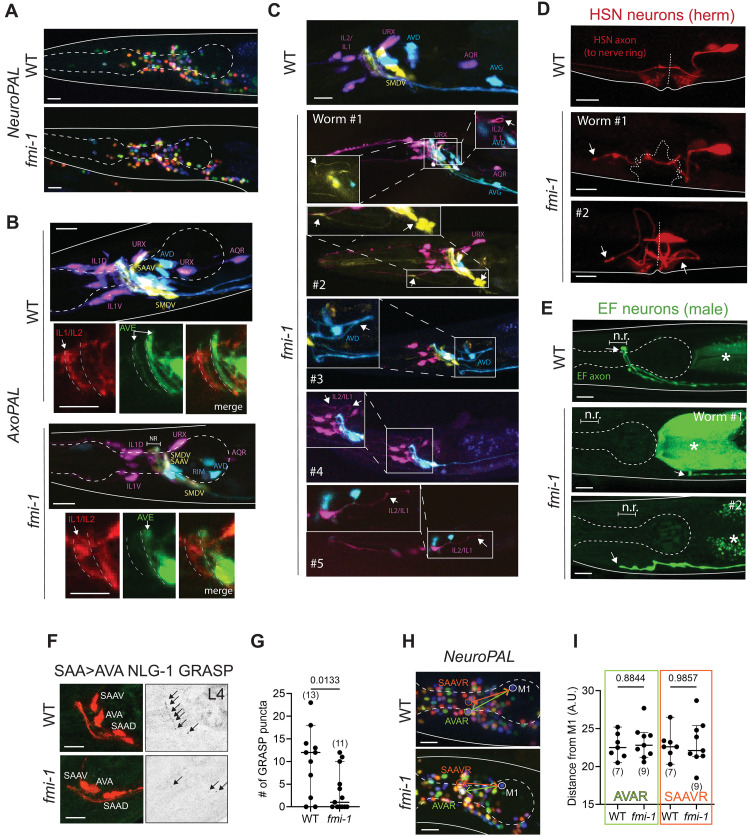
Conserved *fmi-1/Celsr* selectively mediates a subset of neuronal anatomical features. (**A**) Representative images of *NeuroPAL* reporter in the background of *fmi-1* null mutants. (**B**) Representative images of *AxoPAL* reporter in the background of *fmi-1(ot1090)* mutants. (**C**) Representative images of variable axodendritic defects in *fmi-1(ot1090)* mutants assessed with the *AxoPAL* reporter. (**D**) Representative images of variable axon pathfinding defects in hermaphrodite-specific HSN neurons in *fmi-1(ot1090)* mutants. (**E**) Representative images of variable axon pathfinding defects in male-specific EF neurons in *fmi-1(ot1090)* mutants. (**F** and **G**) SAA>AVA synapses are reduced in *fmi-1* null mutants. Representative images (F) and quantification (G) of SAA>AVA NLG-1 GRASP (*otIs839*) puncta in wild-type and *cdh-4(ot1353)* null mutant animals at L1 and L4 stages. Ten animals per genotype were analyzed for the NeuroPAL and AxoPAL analyses. *P* value from unpaired *t* test is shown. (**H** and **I**) AVA and SAA soma positions are unaffected in *fmi-1* null mutants. Representative images (H) and quantification (I) of shortest distance (yellow) between AVAR (green) and SAAVR (orange) soma labeled with *NeuroPAL* (*otIs669*) in wild-type and *fmi-1* null mutant animals at the L4 stage. AVAR and SAAVR were scored relative to M1 pharyngeal neuron, which does not express *fmi-1* and is unaffected in *fmi-1* mutants. *P* values from unpaired *t* test are shown.

The broadly, neuronally expressed FMI-1/CELSR has several neuronal functions in *C. elegans*, as previously described, including axon navigation and synaptogenesis in the ventral nerve cord and sensory dendrite patterning (*33*–*35*, *85*). To expand on the description of *fmi-1/Celsr* function beyond the nerve cord and sensory periphery, we further characterized *fmi-1/Celsr* null mutants in other parts of the nervous system. Using NeuroPAL, we found that neuronal soma positioning was largely unaffected in the head region in *fmi-1/Celsr* mutants (Fig. 8A). In addition, overall nerve ring neighborhood topology was largely unperturbed based on AxoPAL analysis (Fig. 8B). However, a subset of neurons failed to enter the nerve ring (Fig. 8, B to E). For example, in a subset of animals, the AVE axon was missing from the anterior nerve ring neighborhood, which could be due to a defect in AVE neighborhood switch or its failure to enter the nerve ring. Similarly, inner labial neurons IL1/IL2 failed to enter the nerve ring in a subset of animals and their posteriorly projecting axonal loops were found much diverged from the posterior end of the nerve ring (Fig. 8C, worms #1 and #4). Hermaphrodite-specific midbody HSN neurons showed axon pathfinding defects as previously published (*33*), and axons of male-specific EF neurons—whose cell bodies localize in the tail region and axons project to the nerve ring—failed to reach the nerve ring in a manner similar to the HSN phenotype (Fig. 8, D and E). AxoPAL and additional narrowly expressed cytoplasmic reporters revealed additional defects in axodendritic morphologies in *fmi-1/Celsr* mutants. For instance, in a subset of animals, IL1/IL2 neurons altogether lacked axonal loops that typically traverse the width of the nerve ring (Fig. 8C, worm #5).

We also assessed the role of *fmi-1/Celsr* in synapse formation. We have recently shown that *fmi-1/Celsr* is necessary and sufficient for establishing and maintaining neurite contact and proper synapses between neurons PHB and AVA (*85*). Here, we broadened our analysis of *fmi-1/Celsr’s* role in synapse formation as well as other aspects of neuronal architecture. We first used an NLG-1–based GRASP reporter that labels SAA>AVA synapses (*82*), to assess how loss of *fmi-1/Celsr* affects other synapses. We observed a significant reduction in SAA>AVA synapses in *fmi-1(ot1090)* null mutants (Fig. 8, F and G). However, SAA and AVA soma positions as well as their overall morphologies and placement in the nerve ring were unaffected by loss of *fmi-1/Celsr* (Fig. 8, F, H, and I). It is possible that SAA>AVA synapses were lost as a consequence of loss of neurite contact, like in the case of PHB and AVA (*85*). These results, together with the *cdh-4/Fat* analyses described in the previous section, illustrate that different cadherins have distinct roles in nervous system development and that their effects on soma positions and neurite positions can be decoupled from proper synapse formation in the paradigms studied.

### Mapping cadherin expression onto the synaptic connectome

**Fig. 9.**
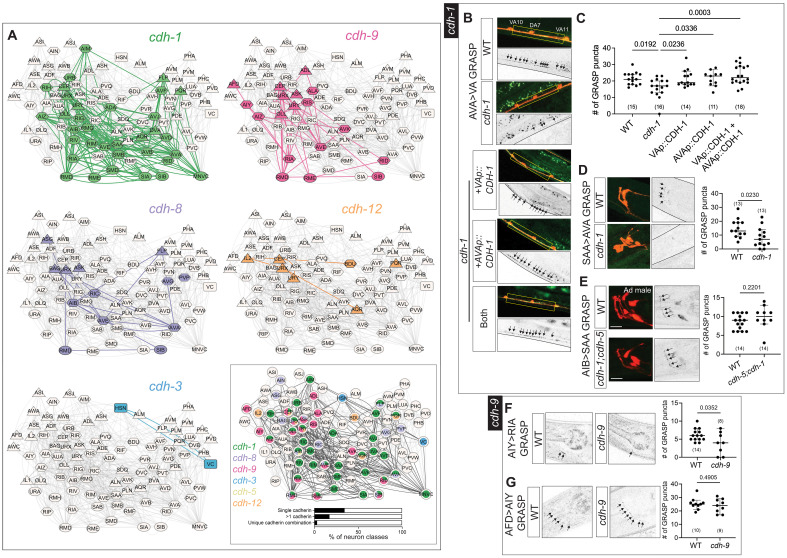
Neurons expressing narrowly expressed cadherins are synaptically connected and promote proper synaptic contacts. (**A**) Expression of *cdh-1*, *cdh-9*, *cdh-8*, *cdh-12*, and *cdh-3* overlaid on the adult connectome (*86*). MNVC includes all ventral nerve cord motor neurons except VC. Boxed panel shows expression of all uniquely and narrowly expressed cadherins overlaid on the adult connectome. Percentages of neuron classes expressing cell-specific cadherins, single or multiple cadherins, and unique cadherin combinations are shown. Triangle, square, and hexagon nodes represent sensory, motor, and interneurons. “Single cadherins” represents the number of neurons expressing only one cadherin, “>1 cadherin” represents the number of neurons expressing more than 1 cadherin, and “unique cadherin combinations” represents neurons with a cadherin repertoire that is not expressed in any other neuron type. (**B** and **C**) AVA>motor neuron (VA) synapses are reduced in *cdh-1(ot1034)* null mutants. Cell-specific expression of full-length CDH-1 cDNA in VA (*otEx8305*), AVA (*otEx8304*), and AVA + VA (*otEx8306*) restores AVA>VA GRASP puncta number. Yellow box represents the site of synapses. *P* values shown were calculated using one-way ANOVA with Bonferroni correction for multiple comparisons. (**D**) SAA>AVA synapses are reduced in *cdh-1(ot1034)* null mutants. (**E**) AIB>SAA synapses are unaffected in *cdh-1(ot1034); cdh-5(ot1093)* double mutants. Representative images and quantification in adult stage males is shown. (**F**) AFD>RIA synapses are reduced in *cdh-9(ot1247)* null mutants. (**G**) AFD>AIY synapses are unaffected in *cdh-9(ot1247)* null mutants. All images, taken of L4-stage animals, are maximum intensity projections of a subset of the Z-stack. Scale bars, 10 μm. In all graphs, a dot represents one worm and error bars denote median with 95% confidence interval. *P* values from unpaired *t* test are shown in (C) to (F).

We next turned from the broadly expressed and conserved cadherins *cdh-4/Fat*, and *fmi-1/Celsr*, to the remaining neuronal cadherins that are all much more restrictively expressed, namely, the conserved cadherins *cdh-1/Dchs* and *cdh-3/Fat,* and the *C. elegans*–specific *cdh-5*, *cdh-8*, *cdh-9*, and *cdh-12* genes (Fig. 2, A to C). We first mapped their nervous system–wide expression onto the *C. elegans* synaptic connectome of the adult (Fig. 9A) (*86*). This mapping is particularly suitable to visualize several points already mentioned above (Fig. 9A): (i) in aggregate, neuron-specific cadherins do not cover the entire nervous system; (ii) there is a finite number of neurons that express multiple cadherins; and (iii) cadherin expression is not biased toward specific parts of the nervous system or does not obviously correlate with specific connectivity features of a neuron. Most importantly, though, such mapping visualizes whether neurons that express a given cadherin are synaptically connected to neurons that express the same cadherin. Because of the well-documented ability of cadherins to engage in homophilic interactions (*6*–*8*), such matching expression may indicate a role in synapse formation or function.

With the obvious exception of *cdh-5*, which is only expressed in a single neuron class, it is indeed apparent that almost all the neuron-type specifically expressed cadherins are expressed in neurons that are synaptically connected to neurons that express the same cadherin (Fig. 9A). To statistically assess whether neurons expressing the same cadherin are more enriched for synaptic connectivity than by chance, we added shuffled controls to compare observed connectivity for cadherins *cdh-1*, *cdh-8*, *cdh-9*, and *cdh-12* against a null distribution (fig. S4A). *cdh-1*–expressing neurons showed a significantly higher observed connectivity proportion than the null model, suggesting that neurons expressing *cdh-1* are indeed more synaptically connected than expected by chance. The observed connectivity proportion for *cdh-8*, *cdh-9*, and *cdh-12* was not significantly different from connectivity in random trials, suggesting that these cadherins are not correlated with synaptic connectivity. Last, taking animal-to-animal variability of synaptic connectivity into account (*87*), we overlaid cadherin expression on a wiring diagram that only contains synaptic connections that are present in all available *C. elegans* datasets of larval-stage and adult animals, of either sex (*88*). In such a “core connectome,” the theme of cadherin-expressing neurons being synaptically connected to neurons expressing the same cadherin still largely holds (fig. S4, B and C).

### Loss of the conserved cadherin *cdh-1/Dchs* and *C. elegans*–specific cadherin *cdh-9* results in synaptic defects

Together with their predicted ability to engage in homophilic protein interactions, the expression of individual cadherins in neurons that are directly connected to one another provides testable hypotheses for potential roles of cadherins in controlling synaptic connectivity and/or synaptic function. We considered cadherins (*cdh-1/Dchs*, *cdh-9*, and *cdh-3/Fat*) for experimental analysis, using previously described GRASP-based markers for synaptic connectivity. Using NeuroPAL and AxoPAL (for *cdh-1/Dchs* and *cdh-9)* or neuron-specific markers (for *cdh-3/Fat)*, we examined neuronal soma position and neurite neighborhood/projections of neurons expressing *cdh-1/Dchs*, *cdh-9*, or *cdh-3/Fat* in the absence of the respective genes and found no obvious defects (fig. S5 and S6A).

On the basis of GRASP marker availability, we examined connectivity of the synaptically connected neuron classes SAA, AVA, and VA, which all express *cdh-1/Dchs* (Fig. 9A). We found a reduction of synaptic puncta in AVA>VA and SAA>AVA synapses trans-synaptically marked with GRASP (Fig. 9, B to D). Restoring CDH-1 separately in AVA and VA neurons, as well as in both AVA and VA, using cell-specific promoters (*ace-2prom3* for VA and *flp-18p* for AVA) to drive full-length CDH-1 cDNA rescued the loss of puncta phenotype observed in whole animal *cdh-1* mutants (Fig. 9, B and C). Synaptic contacts made by SAA to another neuron, AIB, are not affected (Fig. 9E).

In the case of *cdh-9*, we examined its role in specifying synapses between the interconnected, *cdh-9-*positive neurons AFD, AIY, and RIA. GRASP reagents are available to visualize AFD>AIY and AIY>RIA synapses (*83*). In the case of AIY>RIA synapses located in the ventral region of the nerve ring, we observed a reduction in connectivity in *cdh-9* mutants; puncta were fewer and dimmer in mutants compared to wild-type animals (Fig. 9F). AFD>AIY synapses were unaffected in *cdh-9* mutants (Fig. 9G), demonstrating synapse specificity of CDH-9 function, like in the case of CDH-1. Notably, an absence of synaptic phenotype in the AFD>AIY reporter suggests that the overall axonal morphology of AIY neurons is unperturbed in *cdh-9* mutants.

Last, we considered the *cdh-3/Fat* cadherin, which within the nervous system is exclusively expressed in the synaptically interconnected HSN and VC neurons that form the circuit for egg-laying behavior (Fig. 2, A and B) (*89*). Bright HSN and VC expression of *cdh-3/Fat* was detected at the L4/Ad stage, when the egg-laying circuit matures. HSN forms prominent synapses with vulval muscles (Vm) and VC neurons in the vulval region. We visualized HSN>Vm and HSN>VC synapses with trans-synaptic GRASP reporters, but observed no defects in the number or distribution of synaptic puncta in *cdh-3/Fat* mutants (fig. S6, A and B). A marker of presynaptic active zones, CLA-1::GFP, specifically expressed in HSN also showed no defects (fig. S6C); CLA-1 puncta were present and localized properly in the vulval region, in contrast to the ectopic presynaptic specializations observed in other cell adhesion protein mutants (*90*). In addition to vulval synapses, HSN forms synapses with several neuron classes in the nerve ring region (*52*). While we did not test a role of *cdh-3/Fat* in specific nerve ring synapses using GRASP-based reporters, CLA-1::GFP puncta in the nerve ring were unaffected in *cdh-3(ot1035)* mutants (fig. S6D). Because we find *cdh-3/Fat* null mutant to display an egg retention defect akin to (but less severe than) HSN loss (fig. S6E), it is possible that *cdh-3/Fat* may affect synaptic transmission rather than synaptic morphology of the egg-laying circuit. We caution that the expression of *cdh-3/Fat* in other cells of the egg-laying system (Fig. 3B) (*76*) may provide an alternative explanation of the egg retention defects of *cdh-3/Fat* mutants.

### *C. elegans*–specific cadherin CDH-5 is required for proper electric synapse patterning of the AIB interneurons

We discovered a synaptic role for another cadherin, the nematode-specific *cdh-5* gene, which is the only cadherin that is expressed exclusively in a single pair of neurons, the interneuron pair AIB (Fig. 2A and fig. S2B). The neurites of AIB neurons project into the nerve ring, where they generate both chemical and electrical synaptic connections (*52*). Food scarcity and crowded external conditions, prompting animals to enter the dauer diapause state, trigger AIB to generate additional electrical synapses (*65*). Cadherins have been localized to electrical synapses in the vertebrate nervous system (*91*), but it is not known whether cadherins are required for assembling neuronal electrical synapses.

**Fig. 10.**
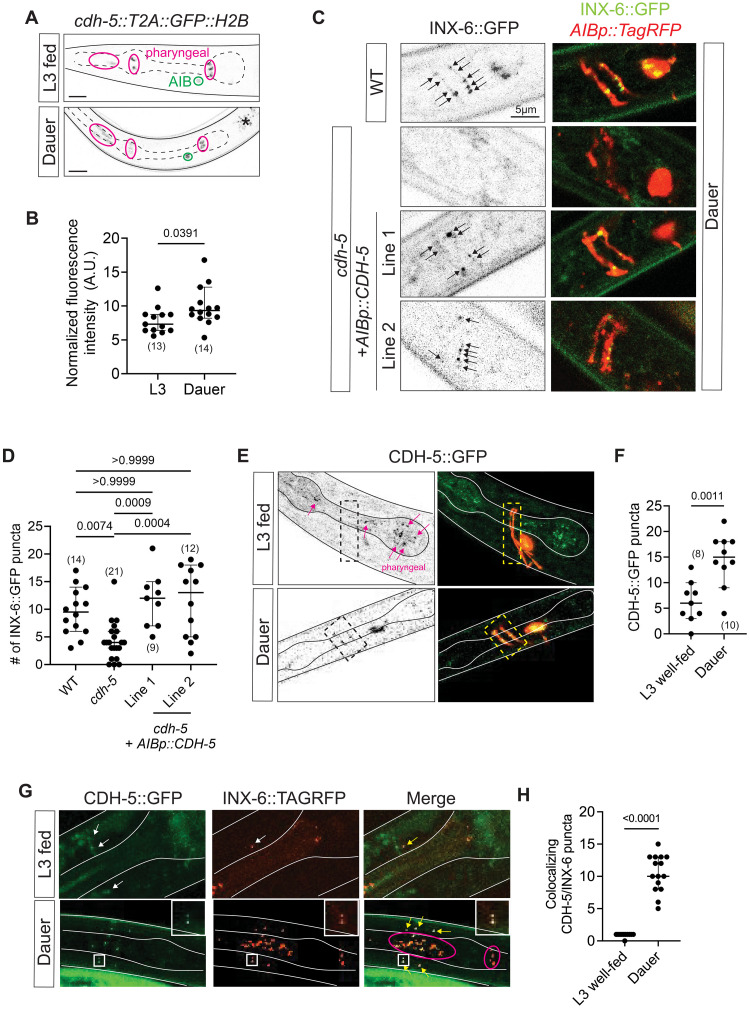
*C. elegans*–specific cadherin *cdh-5* is required for electrical synapse formation in dauers. (**A** and **B**) Experience-dependent plasticity of *cdh-5* expression in AIB neurons. Representative images (A) and quantification (B) of *cdh-5*(*ot1127*) expression in AIB interneurons shows enrichment at the stress-induced dauer stage relative to well-fed L3-stage control. Images denote AIB and nonneuronal *cdh-5* expression in green and pink, respectively. Normalized fluorescence intensity in the AIB neuron closer to the microscope lens in lateral worms is quantified. *P* values from unpaired *t* test are shown. (**C** and **D**) Representative images (C) and quantification (D) of INX-6::GFP (*ot805*) puncta in wild-type and *cdh-5(ot1316)* null mutant dauer worms. Complete loss of CDH-5 leads to a decrease in INX-6::GFP puncta that colocalize with AIB axons. AIB-specific expression of CDH-5 cDNA using *inx-1prom* (*otEx8156*) rescues the loss of INX-6::GFP phenotype in 2 independent transgenic lines. AIB neurons were cytoplasmically labeled with *npr-9p::TagRFP* (*otEx8072*) and only colocalizing INX-6::GFP puncta were scored. *P* values shown were calculated using one-way ANOVA with Bonferroni correction for multiple comparisons. Scale bar, 5 μm. (**E** and **F**) Representative images (E) and quantification (F) of CDH-5::GFP (*syb6640*) puncta localizing on AIB axons labeled with *npr-9p::TagRFP*. Number of CDH-5::GFP puncta increase in dauers relative to L3 well-fed control animals. *P* values from unpaired *t* test are shown. All images are maximum intensity projections of a subset of the Z-stack. Scale bars, 10 μm unless otherwise noted. In all graphs, a dot represents one worm and error bars denote median with 95% confidence interval. (**G** and **H**) Representative images (G) and quantification (H) of colocalizing CDH-5::GFP (*syb6640*) and INX-6::TagRFP (*ot1626*) puncta in dauer and L3 well-fed control animals. Yellow arrows denote colocalizing (inset) CDH-5 and INX-6 AIB puncta in the nerve ring; pharyngeal puncta are marked in pink. *P* values from unpaired *t* test are shown.

Examining *cdh-5* reporter allele expression in dauer stage animals, we found that *cdh-5* is up-regulated in AIB neurons in dauers (Fig. 10, A and B). This up-regulation correlates with the dauer-specific induction of the electrical synapse-forming INX-6 innexin exclusively in the AIB neurons (*65*). Assessment of AIB-localized INX-6::GFP puncta in *cdh-5(ot1093)* null mutants revealed a significant reduction in puncta number in dauer animals relative to wild-type control dauers (Fig. 10, C and D). Restoring CDH-5 specifically in AIB interneurons using an AIB-specific promoter (*inx-1p*) to drive full-length CDH-5 genomic DNA rescued the phenotype of *cdh-5* mutant dauers in two independent transgenic lines (Fig. 10, C and D).

We generated a CRISPR-engineered CDH-5::GFP reporter in which *gfp* is inserted between the PDZ motif-containing C terminus and transmembrane domain to (i) tag all isoforms and (ii) avoid disrupting endogenous protein localization. We observed punctate CDH-5 expression along AIB axons [as well as the pharynx, where INX-6 is also expressed; (*65*)]. In line with dauer-specific up-regulation of our nuclear *cdh-5* reporter, we found an increase in CDH-5::GFP puncta localized on AIB axons in dauer animals (Fig. 10, E and F). To assess colocalization of CDH-5 and INX-6, we used CRISPR-Cas9 to engineer an INX-6::TagRFP reporter allele and found significant overlap of CDH-5::GFP and INX-6::TagRFP puncta in the nerve ring of dauer stage animals (Fig. 10, G and H).

Aside from electrical synapses, we characterized *cdh-5* mutants for defects in AIB soma positions, axon topology in the nerve ring, AIB-specific presynaptic specializations marked with CLA-1::GFP, and specific synapses between AIB and a subset of its foremost chemical synaptic partners (RIM and SAA) labeled with iBLINC and GRASP, respectively, and found no significant phenotypes (fig. S7). These observations indicate that *cdh-5* does not disrupt outgrowth, neighborhood, or chemical synaptic target choice of the AIB axon but apparently has a selective role in promoting electrical synapse formation.

## DISCUSSION

We have provided here a broad, genome- and nervous system–wide view of the expression and function of the cadherin gene family in *C. elegans.* This broad view reveals several themes that we first discuss from the perspective of gene expression patterns and then from the perspective of gene function.

### Dichotomies of cadherin gene expression patterns in the nervous system

Our expression pattern analysis of the entire cadherin gene family, conducted at several stages of development, has revealed several themes.

First, there are two dichotomies in the expression pattern of the 12 *C. elegans* cadherin genes: (i) only two are expressed during proliferative and migratory stages of embryogenesis (classic cadherin *hmr-1* and *cdh-4/Fat*); the expression of all others is only initiated about the time when cells exit the cell cycle and morphogenesis begins; (ii) within the nervous system, 4 of the 12 genes are expressed in an essentially pan-neuronal manner, while six cadherins show a very restricted expression in the nervous system (the remaining two are not expressed at all in the nervous system).

Second, the dichotomy of pan-neuronal and very selectively expressed cadherins almost perfectly correlates with the conservation of cadherins: All four pan-neuronal cadherins are conserved, while all the nonconserved cadherins are very narrowly expressed in the nervous system. There are only two neuron-selectively expressed cadherins that are phylogenetically conserved (*cdh-1/Dchs* and *cdh-3/Fat*).

Third, only a limited fraction of neurons (46%) expresses a neuron type–specific cadherin and expression of the neuron type–specific cadherins shows only limited overlap. Hence, there are only few neuron classes that express unique combinatorial codes of neuron type–specific cadherins (fig. S8).

Fourth, while there are some temporal dynamics of onset of cadherin expression in the embryo, as well as several changes during larval development, all neuronally expressed cadherins show expression in the fully mature, adult nervous system, lasting throughout the life of the animal.

These expression patterns make several predictions about the spectrum of cadherin gene function. First, the broadly expressed cadherins may be involved in “generic,” i.e., non–cell type–specific aspects of cellular morphogenesis or behavior. These generic functions may be implemented in a cell type–specific manner through the interaction of a broad cadherin with cell type–specific partner proteins. Second, expression in the adult nervous system indicates postdevelopmental roles and, third, because there are very few cell-specific combinations of cadherins (fig. S8), it is unlikely that cell-specific cadherin codes define neuron type–specific connectivity features on a broad scale, as they do, for example, in the vertebrate retina (*14*, *21*). Together with the notion that many cells do not even express any cell type–specific cadherin, there does not appear to be a cadherin-based logic that can comprehensively explain synaptic connectivity throughout the entire nervous system of *C. elegans*. While this could be viewed as an overly naïve hypothesis to begin with, one should be reminded that cadherins predate the advent of nervous system and even multicellularity. It therefore appears reasonable to envision that through the expansion of this gene family from a primitive ancestor, cadherin deployment became a pervasively used mechanism for increasing the complexity of cellular interactions in a nervous system. However, at least in *C. elegans*, neuron-specific cadherins only cover parts of the nervous system.

### Cadherin functions on several anatomical scales

Our gene family-wide mutant analysis reveals that the cadherin expression dichotomy correlates with function (table S4). Only two of the *C. elegans* cadherin genes, cadherin *hmr-1* and *cdh-4/Fat,* both phylogenetically deeply conserved, are essential for viability. These are the only cadherins that are already expressed in proliferative embryonic stages during cell focusing and gastrulation. The phenotypes of all other cadherins, including the two pan-neuronal and conserved cadherins *fmi-1/Celsr* and *casy-1/Clstn* are notably subtle on a whole animal level. All animals appear morphologically normal and do not display any gross abnormalities normally associated with strong defects in nervous system development and function, such as the uncoordinated locomotion defects observed upon defects in synaptic transmission and/or failure of the motor system to properly develop (table S3). Behavioral phenotypes for the two pan-neuronal, conserved cadherins *fmi-1/Celsr* and *casy-1/Clstn* only become apparent upon assessing more sophisticated levels of nervous system function, such as complex associative learning paradigms (*38*, *42*–*44*).

Despite the absence of gross behavioral defects, a more detailed anatomical analysis of the consequences of cadherin removal provides a nuanced and panoramic view of cadherin function in *C. elegans,* illustrating that they act on multiple organizational levels and anatomical scales of nervous system development (table S4). Some cadherins appear to function only on some anatomical scales, while others operate on all organizational levels. The latter refers explicitly to the phylogenetically conserved and pan-neuronally expressed *cdh-4/Fat* gene. Previous analysis of *cdh-4/Fat* mutants (*36*, *37*), expanded here by our analysis of *cdh-4/Fat* throughout different parts of the nervous system, using multiple cutting-edge imaging tools and approaches, reveals that *cdh-4/Fat* is required for proper placement of neuronal soma, for proper outgrowth, placement, and relative positioning of neurites within fascicles and for synaptogenesis. At least some of these functions appear to be executed separately from each other, such that defects in synapse formation are not necessarily a consequence of earlier outgrowth defects and, vice versa, that defects in soma positioning do not necessarily lead to defects in synaptogenesis. The phenotypic spectrum of another broadly expressed and conserved cadherin, *fmi-1/Celsr*, is somewhat narrower (i.e., animals are viable and show very limited overall tissue organization defects), but *fmi-1/Celsr* still affects several levels of circuit formation, from axon outgrowth to synapse formation. Such a function of *fmi-1/Celsr* has also recently been delineated in the context of sexual dimorphic maturation of the nervous system (*85*).

The more narrowly expressed cadherins show phenotypes that are limited to specific organizational levels (table S4). Perhaps most interestingly, we observed synaptogenic functions of several conserved and nonconserved cadherins that are accompanied by no obvious defects in other aspects of neuronal morphogenesis. These defects occur in cellular contexts where the respective cadherin is expressed on both the pre- and postsynaptic side of connected cells, implying a homophilic function for these cadherins. Whether these synaptic defects reflect Sperry-type lock-and-key synaptic recognition events or the result of cadherins involved in determining the extent of neurite neighborhood (*88*, *92*) remains to be determined. We note that the effects of cadherins on synaptic readouts are not fully penetrant, indicating that other factors likely play an important role, too.

While functions of cadherins in chemical synapse formation are surely not unprecedented (*17*, *21*, *93*–*96*), we discovered a role of a cadherin, *cdh-5*, in electrical synapse formation. Given the requirement of CDH-5 for proper innexin INX-6 localization and its colocalization with INX-6, we hypothesize that CDH-5 has a direct role in gap junction formation. However, because electron microscopical analysis identified some quantitative changes in adjacency between AIB and partner neurons in dauer versus L3 animals (*87*, *97*) (fig. S9), we cannot exclude the possibility that the primary function of CDH-5 may lie in controlling the extent of membrane contact areas between neighboring neuronal processes to then indirectly promote the establishment of electrical synapses. In either case, it is conceivable that the function for cadherins in localizing innexins may extend to other electrical synapses as well. However, as much as we argue (based on expression patterns) that cadherins are unlikely to play a pervasive role in the formation of all chemical synapses, a broad and invariant role of cadherins in electrical synapse formation appears unlikely as well, again based on cadherin expression patterns, but also based on the notion that the phenotypic spectrum of innexin mutant animals (*98*) is much broader than that of cadherin mutant animals.

### Functions of cadherins in the mature nervous system

One of the most notable aspects of the expression of any *C. elegans* cadherin is their maintained expression in the mature nervous system throughout the life of the animal. Contrasting their well-mapped expression during *Drosophila* and vertebrate embryonic development, cadherin family expression in the mature nervous system has not been comprehensively explored in other organisms. In those cadherin mutants in which we have not observed any obvious developmental or morphological defects, such as in *cdh-3/Fat* mutants, we infer that cadherins may dictate selective aspects of neuron function that is required throughout the life of the neuron. One precedent for such a scenario is the *C. elegans* calsyntenin gene *casy-1/Clstn*, which, in specific cellular settings, is required for synaptic transmission and plasticity, and age-related axon degeneration (*38*, *40*, *42*–*44*, *99*). Functions of several vertebrate cadherins in synaptic signaling, homeostasis, and maintenance have been described (*100*) and we consider those to be the most likely functions of the *C. elegans* cadherins for which we have not found a function yet.

### Similarities and dissimilarities between the function of animal cadherins

There are notable parallels of *C. elegans* and vertebrate cadherin function, but besides pointing to novel aspects of cadherin function (e.g., in electric synapse formation), our analysis also reveals notable differences. First and foremost, on the level of existence of genes, there are no protocadherins in *C. elegans*. In vertebrates, protocadherins are thought to be involved in self-avoidance of sister neurites of a developing neuron (*45*, *101*). The much less elaborated nature of *C. elegans* neurites (*52*) is a possible explanation for why protocadherins do not exist in *C. elegans.*

Several of the cadherins conserved between worms and vertebrates have similar functions. For example, there are common roles of FMI-1 and its vertebrate homolog CELSR in axon pathfinding and synaptogenesis (*33*, *35*, *85*, *102*–*104*) and CDH-4 and its vertebrate homolog FAT in neuronal migration and axon pathfinding (*36*, *37*, *105*–*109*). On the other hand, there are also apparent functional discrepancies that may point to novel and currently unexplored aspects of vertebrate cadherin function. For example, in both *Drosophila* and vertebrates, FAT-type cadherins operate together with a DCHS/Dachsous-type cadherin as a ligand/receptor pair to fulfill a number of diverse roles during morphogenesis and embryonic pattern formation, within and outside the nervous system (*105*, *106*). Yet, in *C. elegans*, we found that loss of the sole Dachsous ortholog, *cdh-1* (which had not been studied before), results in none of the obvious neuronal developmental and morphological defects of *cdh-4/Fat* mutants. Moreover, unlike *cdh-4/Fat* mutants, *cdh-1/Dchs* mutants are completely viable. This lack of matching phenotype is also consistent with the notably more restricted expression of *cdh-1/Dchs.* These observations strongly argue for FAT proteins having DCHS-independent function, possibly as homophilic adhesion molecules or via binding partners that are unexplored in any animal system (*105*).

In conclusion, by taking a panoramic genome- and animal-wide approach, we identify roles of cadherins in novel cellular contexts. We conclude that cadherin functions at various anatomical scales (animal morphogenesis, behavior, soma positions, axon patterning, and synapses) to orchestrate the development of some components of the *C. elegans* nervous system. Our analysis was largely focused on differentiated neuronal features, but cadherins have been shown to play important roles in several nonneuronal contexts (*108*, *110*, *111*), and our comprehensive expression atlas provides an entry point for exploring cadherin function more deeply and in additional contexts, such as in nonneuronal tissues. In addition, our work provides a resource for exploring additional neuronal roles of cadherins in synapse maintenance and experience-dependent behavioral plasticity, axon repair, or during sexually dimorphic nervous system maturation.

### Limitations of the study

We note several limitations of our expression pattern analysis. First, although our gene expression atlas is largely in concordance with scRNA-seq data, the expression reporters described here may not represent levels of protein expression, confounding interpretation of expression and connectivity correlations. Given previous work showing the functional significance of varying cadherin levels (*26*), it is difficult to interpret differences in expression levels in our reporters and, thus, we treat expression as binarized for the bulk of our analyses. In addition, the reporters are designed to drive GFP expression into the nucleus for ease of scoring in the background of the landmark reporter NeuroPAL. However, this excludes capturing the diversity of subcellular localization patterns of cadherin proteins, which may be revealing of cadherin function. An exception to this is CDH-5, where our translational reporter shows subcellular localization along AIB axons and colocalization with gap junction protein INX-6. In addition, the quantitative *cdh-5* expression difference observed between well-fed L3 and dauer animals is recapitulated by our protein reporter, where we see an increase in puncta in dauers (Fig. 10).

Another limitation pertaining to the mutant analysis is that we assay a limited number of cadherin-expressing neurons for synapse phenotypes; despite the breadth of the analysis presented, the absence of phenotypes in synapses assayed does not exclude the possibility of phenotypes in other synapses. In addition, the reporters used to visualize chemical synapses are a proxy for synapse structure and do not necessarily present the ground truth for submicrometer-resolution synapses. All GRASP reporters used are based on localization of the synaptic protein NLG-1, and hence, the reconstituted GFP signal categorically represents NLG-1 localization. In construction of GRASP reporters, we tried to mitigate technical variability stemming from overexpressed multicopy transgenic arrays, as described previously (*82*), but these reporters may still occasionally lead to an under- or overrepresentation of synaptic signal. Validating phenotypes with reporters that endogenously label neuron-specific pre- and postsynaptic proteins, as we did in the case of *fmi-1* in a previous study (*85*), or are single-copy transgenes represents an important next step.

## MATERIALS AND METHODS

### *C. elegans* strains and maintenance

Worms were grown at 20°C on nematode growth media (NGM) plates seeded with *Escherichia coli* (OP50) bacteria as a food source, as previously described (*112*). Worms were maintained according to standard protocol. The wild-type strain used in this study denotes the *C. elegans* Bristol variety (N2). A complete list of strains used in this study is listed table S5. Strains generated in this study are deposited at the CGC.

### CRISPR-Cas9–based genome engineering

To generate endogenously tagged T2A::GFP::H2B cadherin reporters as previously described (*113*), the following sgRNAs were used:

*cdh-1*: 5′AACGATGGAATGATTGGTGG3′.

*cdh-3*: 5′GCCCCATCTTACCGTAGAGA3′.

*cdh-5*: 5′CGTTGATGATAATCTGGTGT3′.

*cdh-8*: 5′GACTGCAAATCTGCAGAAGT3′.

*cdh-9*: 5′acataTTAAAAATAGACAGT3′.

*cdh-12*: 5′CATCGTTGGCAATTGATGGG3′.

*casy-1*: 5′AGAACGAGCGTTCGTTGAGA3′ and 5′GCAAATCAACGTGTCGTTGG3′.

To generate full locus cadherin deletion alleles, two guide sgRNAs at either end of the locus were used in a single injection as previously described (*114*), as follows:

*cdh-1(ot1034* and *ot1299)*: 5′TTGGGAACATGATGTTTCGG3′ and 5′AACGATGGAATGATTGGTGG3′.

*cdh-3(ot1102* and *ot1248)*: 5′CGAAAACTTGATCGTCTCTT3′ and 5′GCCCCATCTTACCGTAGAGA3′.

*cdh-3(ot1035)*: 5′CGAAAACTTGATCGTCTCTT3′ and 5′CTTCTATGAAAATAGTTGCA3′.

*cdh-4(ot1246* and *ot1353)*: 5′AGTCACCACATCTTCTTCTA3′ and 5′GTCTGTGAATCTAATGAGAT3′.

*cdh-5(ot1093)*: 5′TGTTTCAGTTAAGAACCCTT3′ and 5′CGTTGATGATAATCTGGTGT3′.

*fmi-1(ot1090)*: 5′TTGAATGTGAATGTCAGTGG3′ and 5′TGATGCGTATTACACATATA3′.

*cdh-8(ot1084* and *ot1319)*: 5′GGTGTCTAAAAGGAACAGGT3′ and 5′GACTGCAAATCTGCAGAAGT3′.

*cdh-9(ot1247* and *ot1300)*: 5′TAGTAAGGATCGGCCCAAGT3′ and 5′ACATATTAAAAATAGACAGT3′.

*cdh-12(ot1091)*: 5′CTGCGAATAATAGTATGAAG3′ and 5′CATCGTTGGCAATTGATGGG3′.

*casy-1(ot1082)*: 5′AGCATGGTGATGTTTGGCGT3′ and 5′GCAAATCAACGTGTCGTTGG3′.

To generate *unc-86* deletions *ot1355* and *ot1354* in the background of *cdh-12(ot1119)* and *fmi-1(syb4563)* GFP reporters, respectively, the following sgRNAs and ssODN were used:

sgRNA 1: 5′CAAGGTCCCCCTCTTTTCCA3′.

sgRNA 2: 5′ACAACATACAATGGGCTACC3′.

Repair template: 5′TCTGTCTCCTCCCAGCTTCAAGGTCC-CCCTCTTTTACCTTGATTCTTTGATTAGTTTCGTTTT-CGTGAAC3′.

To generate *lin-29(ot1482)*, sgRNA 5′TCTTTTTTACCTCAGT-TCTT3′ and custom TagRFP ssODN were used. CRISPR-tagged SL2::GFP::H2B reporters for *fmi-1(syb4563)*, *hmr-1(syb4454)*, *cdh-4(syb4476)*, and *cdh-7(syb4675)* were made by SunyBiotech. The CDH-4::GFP CRISPR reporter (*syb6764*) was also made by SunyBiotech.

### Molecular cloning

AxoPAL:Transgenic reporter *otEx7895* (AxoPAL v1.0) contains the following reporters: nmr-1::CyoFP (AVA, AVE, AVD, RIM, AVG, and PVC), flp-3::mNeptune (IL1), gcy-35::mNeptune (URX, AQR, ALN, PQR, PLN, and SDQ), flp-7::tagRFP (SAA), klp-6::tagRFP (IL2), and C42D4.1::GFP (RMD and SAA). Promoters were cloned using standard restriction digestion cloning or excising NLS/H2B sequence from plasmids used to generate NeuroPAL (*29*). All plasmids were coinjected in N2 strain at 30 ng/μl each; no coinjection marker was used because the cytoplasmic reporter expression was visibly bright. Transgenic worms were maintained by picking fluorescent worms.

Primers for respective gene promoters used in AxoPAL v1.0 are as follows:

*nmr-1p*—forward: gaaatgaaataAGCTTGCATGCCTGCAGctgctgctgtaggctttg, reverse: GTCCTTTGGCCAATCCCGGGatctgtaacaaaactaaagtttgtcg; 2×NLS and H2B were excised from pEY32 [Addgene # 191037, (*29*)] using Gibson.

*gcy-35p*—forward: gaaatgaaataAGCTTGCATGCCTGCagtttccgcatatagcttatag, reverse: GTCCTTTGGCCAATCCCGGGattctactctccgcaaaaaagtaac; 3×NLS was excised from plasmid pEY45 [Addgene # 191049, (*29*)] using Gibson.

*klp-6p* (pCC045)—forward: cgttttggagtttgctacga, reverse: tattctgaaaagttcaactaataaatttag.

*flp-3p*—forward: gaaatgaaataAGCTTGCATGCCTGCAGACTAgttccgcatggaataatagcc, reverse: GTCCTTTGGCCAATCCCGGGtggtggttatggtggtgttac; 3×NLS was excised from pEY44 [Addgene # 191048, (*29*)] using Gibson.

*flp-7p*—forward: actgttgcttggtcttgtg, reverse: ttctaaaagtctttgaatgaaaacgag.

*C42D4.1p* [pCC277, (*49*)].

*cdh-5* rescue: Genomic *cdh-5* sequence was amplified from wild-type N2 lysate and cloned into *inx1p::SL2::TagRFP* plasmid using Gibson cloning (NE Builder HiFi DNA Assembly Master Mix, E2621L).

*cdh-1* rescue: To generate pCPL26(*flp-18p:: cdh-1(cDNA)::SL2::3xNLS::GFP*), *cdh-1* cDNA was amplified from N2 cDNA, the SL2-backbone was amplified from pCPL3(*srab-20p::lin-29a::SL2::3XNLS:*: *GFP*), and *flp-18p* (~1.4 kb) was amplified from pCPL17(*flp-8p::3XNLS::Cre*). The three fragments were ligated via Gibson cloning (NEBuilder HiFi DNA Assembly Master Mix). To generate pCPL27(*ace-2prom3p::cdh-1(cDNA)::3xNLS::GFP*), the VA-specific promoter fragment was synthesized (AZENTA life sciences) and subcloned into pCPL26(*flp-18p:: cdh-1(cDNA)::SL2::3xNLS::GFP*).

Cell-specific *cdh-4* RNAi: To generate *pCPL28(flp-18p::ds cdh-4 RNAi (+)::SL2::3xNLS::GFP)* and *pCPL29(flp-18p::ds cdh-4 RNAi (−)::SL2::3xNLS::GFP)*, the *cdh-4* double-stranded RNAi fragment was amplified with 5′-TGATACCGTGGATGATGTTCTAGCAAC-3′, 5′-AAAGCTTGTACATAGAATGAATATTCCTTC-3′, 5′-AAAGCTTGTACATAGAATGAATATTCCTTCATC-3′, 5′-AAAGCTTGTACATAGAATGAATATTCCTTCATC-3′, and 5′-TGATACCGTGGATGATGTTCTAGCAAC-3′, respectively, from N2 genomic DNA and the SL2-backbone amplified from pCPL26(*flp-18p:: cdh-1(cDNA)::SL2::3xNLS::GFP*). To generate *pCPL30(srab-20p::ds cdh-4 RNAi (+)::SL2::3xNLS::GFP)* and *pCPL31(srab-20p::ds cdh-4 RNAi (−)::SL2::3xNLS::GFP)*, *srab-20p* was digested from pCPL3(*srab-20p::lin-29a::SL2::3XNLS:: GFP*) by Sph I and Xma I restriction enzymes and subcloned into pCPL28 and pCPL29, respectively.

### Expression analysis and cell identification

L4/Adult neuronal identification (ID): Cell-specific neuron ID for all cadherin reporters was performed by colocalization with the NeuroPAL landmark reporter transgene (*otIs669*) as described previously (*29*). Briefly, each cadherin reporter strain was crossed into NeuroPAL strain, and 10 to 15 animals were imaged for analysis. Animals were imaged in the lateral position to facilitate ID. For L1 scoring, some animals were imaged in a dorsoventral position to facilitate neuronal ID in crowded ganglia. Synchronized L1- and L4-stage animals were obtained by egg prep.

Developmental neuronal ID: Transient cadherin expression was identified by first identifying expression at L4 stage for ease of scoring. Sequentially working backwards, expression was then identified at stages L3, L2, and L1 to reduce the number of neurons to be identified at L1.

Nonneuronal ID: For nonneuronal cell ID, cadherin-positive cells were identified by assessing a combination of features including position, subcellular features based on Nomarski optics (*115*), and non-overlap of GFP with the pan-neuronal TagRFP transgene in NeuroPAL. In both neuronal and nonneuronal cells, expression was scored as present or absent, irrespective of strength of the fluorescent signal, if it was consistent across 5 to 10 animals.

### Cadherin expression and connectivity analysis

For each cadherin, to statistically assess whether neurons expressing that cadherin are more enriched for synaptic connectivity than would be expected by chance, we first derived the null distribution of connectivity proportion by generating pseudo-connectomes. This was done by shuffling synaptic connectivity in a subset of randomly chosen neurons (the number of random neurons was equivalent to the number of neurons expressing that cadherin in our expression atlas); shuffling obeyed topological constraints, i.e., only physically adjacent neurons, based on EM-reconstructed adjacency, were permitted to be synaptically connected. We performed this shuffling over 1000 trials to derive the null distribution of connectivity and compared this null distribution to the observed connectivity proportion for that cadherin. Connectivity proportion was calculated by counting the number of synaptically connected neuron pairs and dividing by the total number of all possible pairs. *P* value was computed to assess the proportion of random trials where the connectivity proportion exceeded the observed value. This analysis was performed in R.

### Automated worm tracking

Crawling assay: Crawling locomotion features were analyzed using an automated multiworm tracker system (MBF BioScience) and WormLab software (MBF BioScience) (*77*). Briefly, five to eight L4 hermaphrodite animals were placed on 5-cm NGM plates and videos were recorded for 4 min at room temperature. Videos were captured at 7.7 μm/pixel camera resolution to largely cover a 5-cm plate (region of interest)*.* NGM plates were fully coated with a thin lawn of OP50 16 to 18 hours before the assay to prevent bacterial overgrowth and avoid biased locomotion at the edge of the bacterial lawn. L4 animals were synchronized via egg prep, and well-fed animals were used for tracking. Mutant and wild-type N2 control animals were tracked on the same day by alternating between the two genotypes. Altogether, 10 to 15 animals were used for each condition.

Swimming assay: Swimming assay was performed as previously described using the WormLab automated multiworm tracker (MBF BioScience) (*77*). Briefly, five L4 hermaphrodite animals, synchronized via egg prep, were transferred to 50 µl M9 buffer and recorded for 1 min. Quantitative analysis of swimming locomotion was performed using the WormLab software (MBF BioScience). Worms that did not move right after placing in M9 were discarded from further analysis. Animals from multiple plates were pooled and population-level swimming features—including speed, wave initiation rate, activity index, and curling—as described previously (*77*) were analyzed for statistical significance.

### Dauer selection

For *cdh-5* analyses, dauers were induced under starvation, crowding, and high-temperature conditions. Briefly, 10 L4 hermaphrodite animals were placed on regular 5-cm NGM plates seeded with *E. coli* (OP50) and incubated at 25°C. After 1.5 weeks, the plates contained a mixed population of dauers and non-dauers. To isolate enough dauers for analysis, SDS selection was performed as previously described (*116*). Briefly, a mixed population of animals was washed off plates, centrifuged, resuspended in 1% SDS, and incubated at room temperature with gentle agitation. After 30 min, dauer larvae were centrifuged, washed twice with M9 to remove SDS, and transferred to uncoated NGM plates. All experiments were performed within 1 hour of transfer to prevent animals from exiting dauer arrest. To compare dauer animals to L3 well-fed wild-type N2 control, L3 animals were synchronized via egg prep.

### Microscopy

Worms were anesthetized using 100 mM sodium azide (NaN) and mounted on glass slides with 5% agarose pads. Images were acquired using a confocal laser scanning microscope (Zeiss LSM880) and analyzed using ZEN Imaging software (blue edition, Carl Zeiss) or Fiji ImageJ (*117*). NeuroPAL and AxoPAL images were acquired at 40×. Synaptic reporter images were typically acquired at 63×.

### Statistical analysis

All statistical analysis was performed using Prism (GraphPad). Details of analyses are mentioned in figure legends. For tracking analyses, locomotion metrics were first exported from WormLab to Excel, and statistical tests were subsequently performed in Prism.

### Analysis of AxoPAL neighborhood topology

Axonal topology was visualized in Fiji by first cross-sectionally slicing the region of the nerve ring and max-projecting the resliced region onto the *Z* plane in increments spanning the nerve ring width to assess nerve ring composition along the anterior-posterior axis.

### Quantification of cell body positions

To score cell body positions in the anterior ganglion for assessing soma displacement in *cdh-4* mutants, we used the NeuroPAL ID software to get *x*, *y*, and *z* coordinates of each neuron (*29*). For each neuronal pair, the Euclidean distance was computed to build a distance matrix in Matlab.

### Quantification of synaptic puncta

ASK>AIA, PHB>AVA, BDU>HSN, SAA>AVA, and AIB>SAA GRASP puncta were scored using the semiautomated worm puncta scoring software WormPsyQi (*82*). In all cases, a neurite mask was not used for synapse segmentation except in the case of AIB and NSM CLA-1 datasets.

AIB>RIM iBLINC puncta were scored by first cross-sectionally slicing the region of the nerve ring containing iBLINC puncta, making it in the *Z* plane, and then counting manually in Fiji. AFD>AIY, AIY>RIA, HSN>Vm, and HSN>VC were scored using the cell counter plugin in Fiji.

CLA-1 puncta were scored for overall number and puncta features—such as mean pixel intensity and mean inter-synapse distance—using WormPsyQi.

### Quantification of PHB and AVA contact

Contact site length between PHB and AVA processes in the PHB/AVA CD4 reporter was quantified in Fiji ImageJ (*117*). Briefly, the entire Z-stack was scanned while tracing over the GFP^+^ region (where the PHB and AVA processes overlap) with a segmented line and then measuring the overall line length. In cases where the contact and resulting GFP signal was discontinuous, multiple lines were drawn, measured independently, and summed to yield the overall contact site length. For visualization purposes, figures contain a representative subset of Z-stacks reconstructed as maximum intensity projection using the Zen Imaging software (blue edition, Zeiss) to display the maximal PHB/AVA contact site.
