# Supplementary material for: Nervous system–wide analysis of all C. elegans cadherins reveals neuron-specific functions across multiple anatomical scales
Source: Sci Adv. 2025 Feb 21;11(8):eads2852. doi: 10.1126/sciadv.ads2852 (PMC11844738; doi:10.1126/sciadv.ads2852)
Supplement: Supplementary file 1 — Figs. S1 to S9 Legends for tables S1 to S5 [file sciadv.ads2852_sm.pdf]

Science Advances
AAAS

Supplementary Materials for

Nervous system–wide analysis of all *C. elegans* cadherins reveals neuron-specific functions across multiple anatomical scales

Maryam Majeed *et al.*

Corresponding author: Oliver Hobert, or38@columbia.edu

Sci. Adv. **11**, eads2852 (2025)
DOI: 10.1126/sciadv.ads2852

The PDF file includes:

Figs. S1 to S9
Legends for tables S1 to S5

Other Supplementary Material for this manuscript includes the following:

Tables S1 to S5

Figure S1

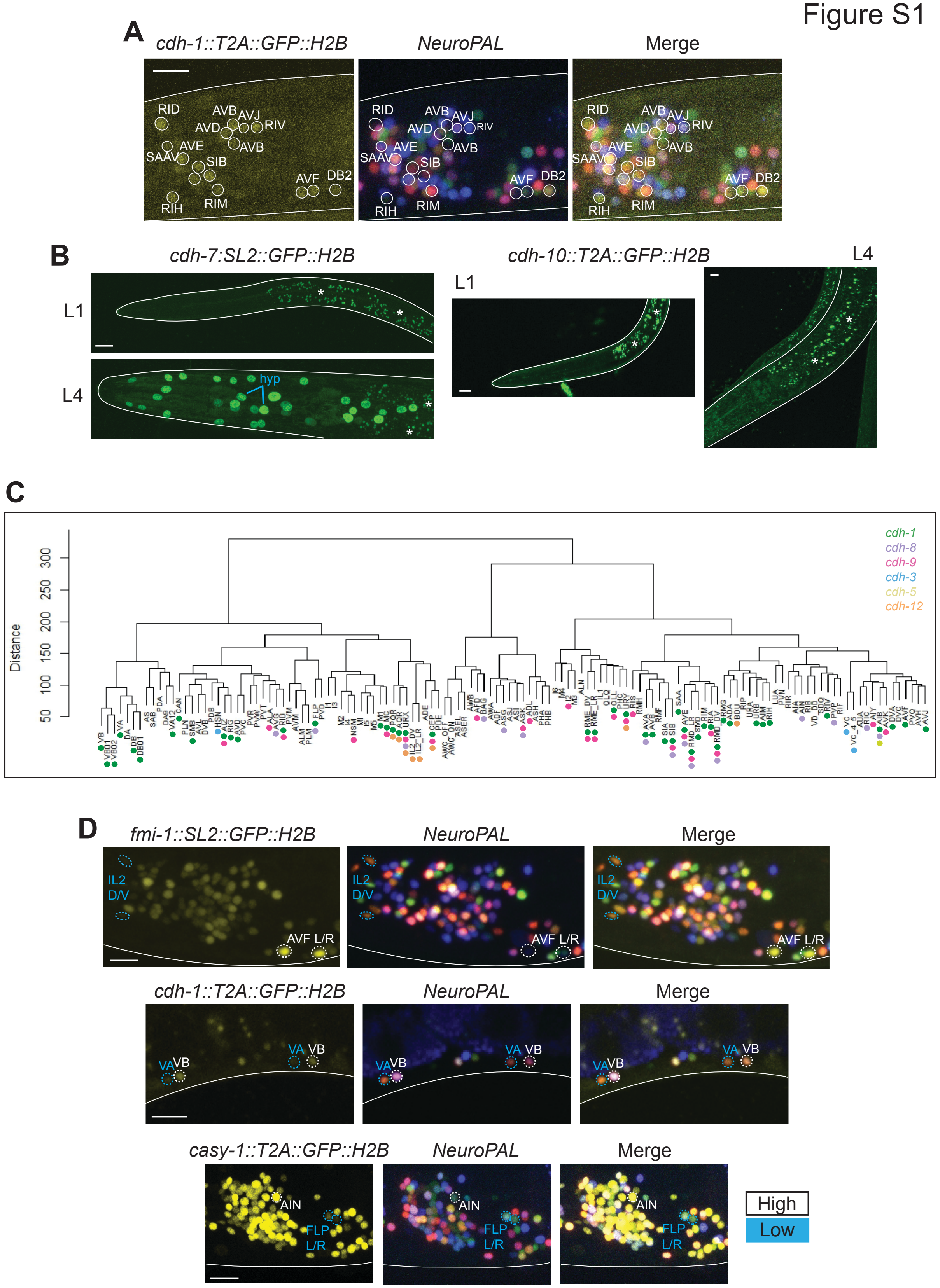
A
cdh-1::T2A::GFP::H2B
NeuroPAL
Merge
RID
AVB
AVJ
AVD
RIV
AVE
SAAV
SIB
AVF
DB2
RIH
RIM
B
cdh-7:SL2::GFP::H2B
cdh-10::T2A::GFP::H2B
L1
L4
hyp
C
Distance
cdh-1
cdh-8
cdh-9
cdh-3
cdh-5
cdh-12
D
fmi-1::SL2::GFP::H2B
IL2 D/V
AVF L/R
VA
VB
casy-1::T2A::GFP::H2B
AIN
FLP L/R
High
Low

Figure S1: Cellular expression analysis of cadherin reporters

(A) Representative images of the *cdh-1::T2A::GFP::H2B* reporter (*ot1092*) showing cell-specific identification of neurons based on overlay with the landmark reporter NeuroPAL (*otIs669*). Images are of L4-stage animals and represent maximum intensity projections of a subset of the Z-stack.

(B) Representative images of reporters *cdh-7::SL2::GFP::H2B* (*syb4675*) and *cdh-10::T2A::GFP::H2B* (*ot1117*). Gut autofluorescence is marked with an asterisk.

(C) Dendrogram showing neurons clustered by genetic similarity. Cadherin expression for narrowly-expressing cadherins is denoted in different colors.

(D) Differential expression levels for the same cadherin type across neuron types. Representative images of *fmi-1::SL2::GFP::H2B* (*syb4563*), *cdh-1::T2A::GFP::H2B* (*ot1092*), and *casy-1::T2A::GFP::H2B* (*ot1108*) across representative neuron types with low (blue) or high (white) expression.

Scale bars = 10µM.

Figure S2

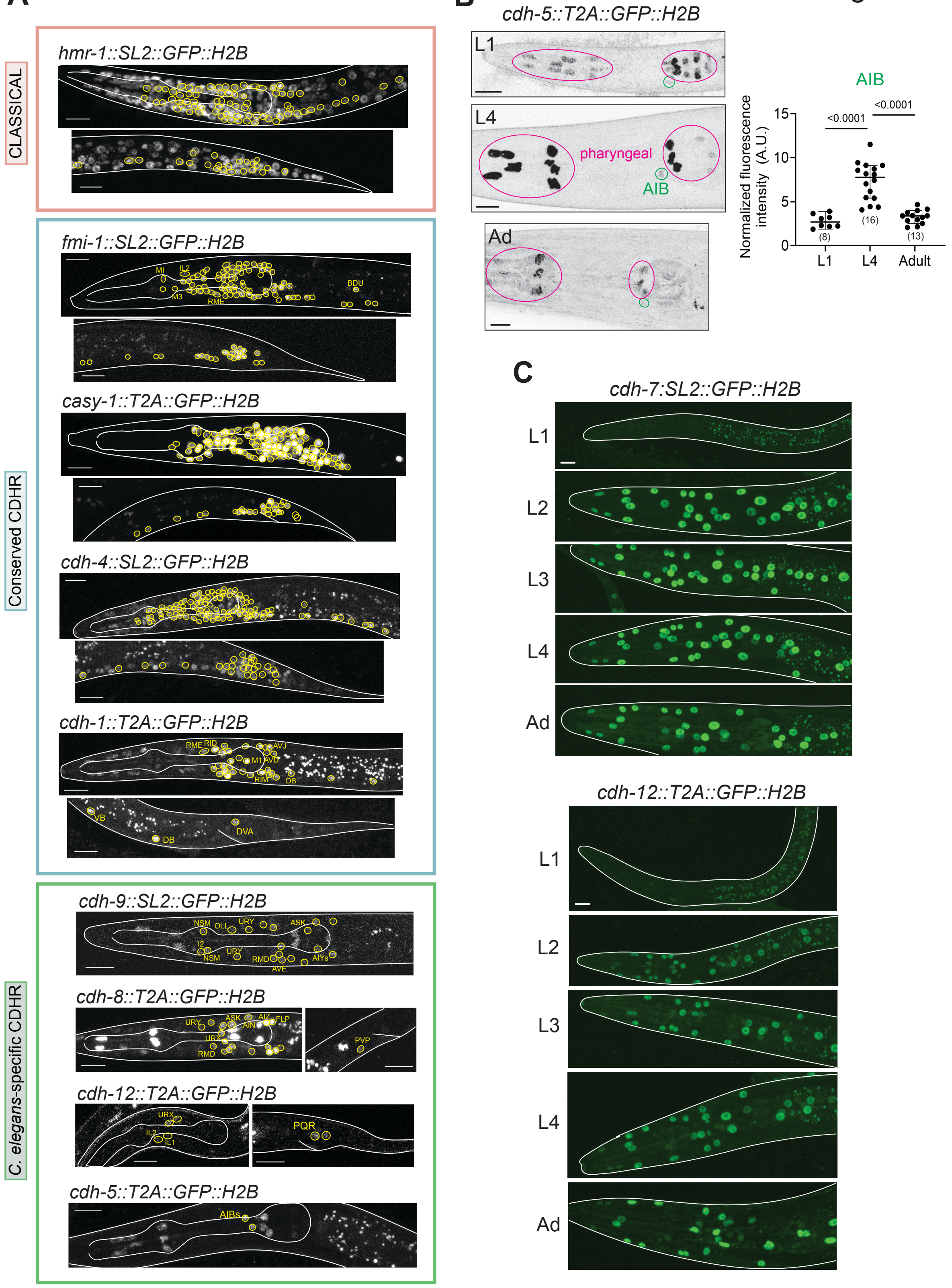

A
CLASSICAL
hmr-1::SL2::GFP::H2B
Conserved CDHR
fmi-1::SL2::GFP::H2B
MI
IL2
M3
RME
BDU
casy-1::T2A::GFP::H2B
cdh-4::SL2::GFP::H2B
cdh-1::T2A::GFP::H2B
RME RID
AVJ
M1 AVD
RIM
DB
VB
DB
DVA
C. elegans-specific CDHR
cdh-9::SL2::GFP::H2B
NSM
OLL
URY
ASK
I2
NSM
URY
RMD
AVE
AIYs
cdh-8::T2A::GFP::H2B
URY
ASK
AIZ
FLP
AIN
URX
RMD
PVP
cdh-12::T2A::GFP::H2B
URX
IL2
IL1
PQR
cdh-5::T2A::GFP::H2B
AIBs
B
cdh-5::T2A::GFP::H2B
L1
L4
pharyngeal
AIB
Ad
AIB
Normalized fluorescence intensity (A.U.)
15
10
5
0
<0.0001
<0.0001
(8)
(16)
(13)
L1
L4
Adult
C
cdh-7:SL2::GFP::H2B
L1
L2
L3
L4
Ad
cdh-12::T2A::GFP::H2B
L1
L2
L3
L4
Ad

Figure S2: Cadherin expression in early development

(A) Representative images of cadherin expression in L1 animals. Reporters of classical cadherin *hmr-1*(*syb4454*) (pink) and non-classical cadherins (green) *casy-1*/*Clstn* (*ot1108*), *cdh-4*/*Fat* (*syb4476*), and *fmi-1*/*Celsr* (*syb4563*) are pan-neuronally or broadly expressed. Reporters of remaining cadherins *cdh-1*(*ot1092*), *cdh-3*(*ot1096*), *cdh-5*(*ot1127*), *cdh-8*(*ot1106*), *cdh-9*(*ot1095*), and *cdh-12*(*ot1119*) are relatively sparsely expressed. All GFP+ neurons are encircled in yellow. Images are of L1-stage animals and represent maximum intensity projections of a subset of the Z-stack. Scale bars = 10μM.

(B) Developmental changes in *cdh-5(ot1127)* expression.

(C) *cdh-7*(*syb4675*) and *cdh-12*(*ot1119*) expression onset in the hypodermis begins at L2 and persists until adulthood.

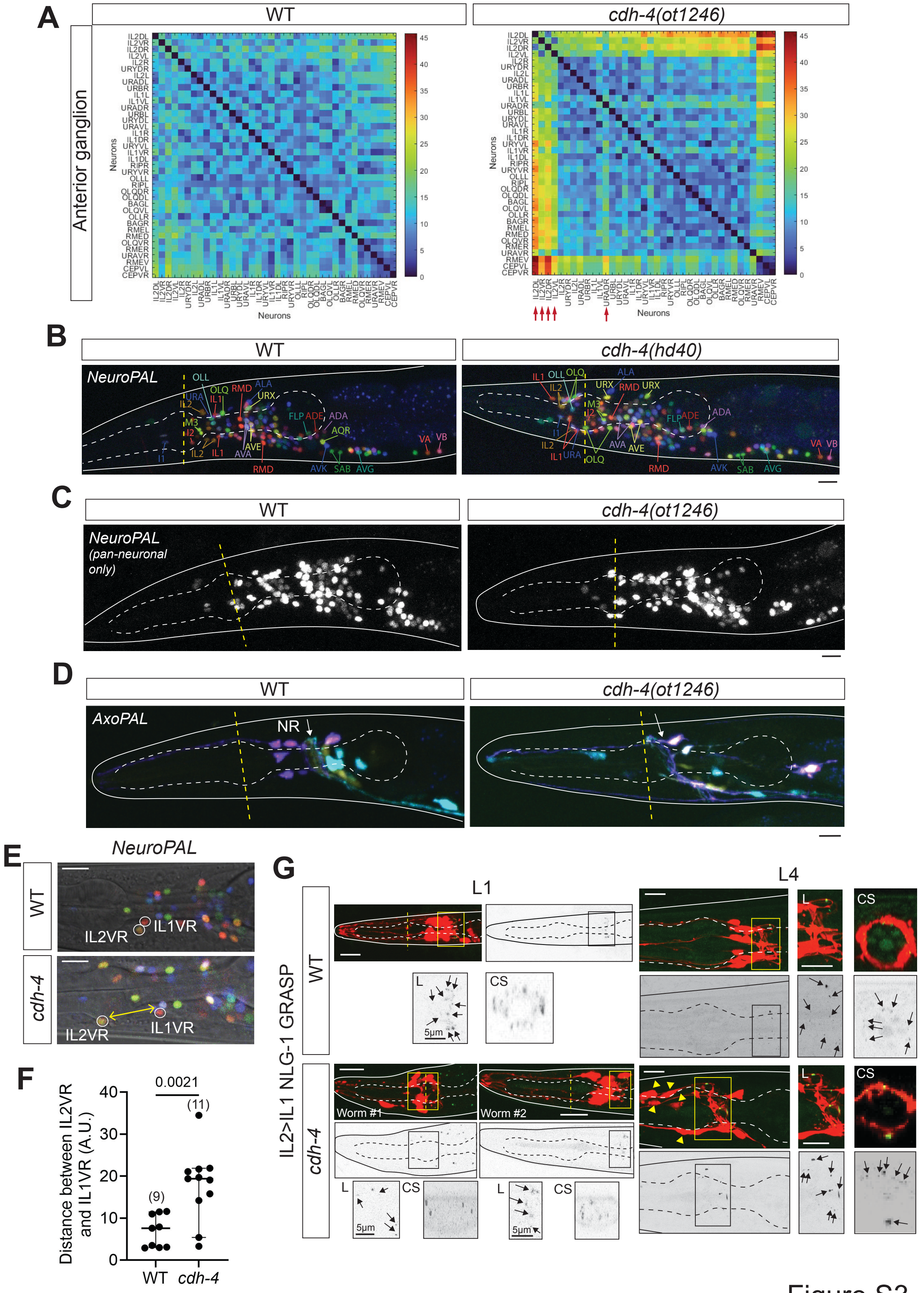
A
WT
cdh-4(ot1246)
Anterior ganglion
Neurons
B
WT
cdh-4(hd40)
NeuroPAL
C
WT
cdh-4(ot1246)
NeuroPAL
(pan-neuronal
only)
D
WT
cdh-4(ot1246)
AxoPAL
NR
E
NeuroPAL
WT
cdh-4
IL2VR
IL1VR
F
Distance between IL2VR
and IL1VR (A.U.)
0.0021
(9)
(11)
WT
cdh-4
G
IL2>IL1 NLG-1 GRASP
L1
L4
WT
cdh-4
Worm #1
Worm #2
L
CS
5μm

Figure S3

Figure S3: Phenotypic analysis of *cdh-4* mutants

(A) Distance matrices showing pairwise distance between neurons in the anterior ganglion in WT and *cdh-4(ot1246)* mutants. Neurons displaced anteriorly are marked in red arrows (n=5 per genotype).

(B) Neuronal soma mispositioning defects in *cdh-4*(*hd40*) mutants phenocopy defects in *cdh-4(ot1246)* mutants as assessed with NeuroPAL (*otIs669*).

(C) Neuron number is unaffected in *cdh-4(ot1246)* mutants, as assessed with a pan-neuronal reporter in the NeuroPAL (*otIs669*) landmark strain.

(D) Nerve ring anterior shift in *cdh-4(ot1246)* mutants as visualized with AxoPAL (*otEx7895*) reporter.

(**E, F**) IL2 and IL1 soma are mispositioned in *cdh-4(ot1246)* mutants. Representative images (D) and quantification (E) of shortest distances (yellow) between IL2VR and IL1VR soma labeled with *NeuroPAL* (*otIs669*) in wild type and *cdh-4* mutant animals at the L4 stage. P-value from unpaired t-test is shown.

(**G**) IL2>IL1 synapses labeled with a GRASP reporter (*otIs657*) can still be observed in *cdh-4(hd40)* mutants at L1 and L4 stages. *cdh-4* L1 images show 2 representative worms with variable IL1/IL2 soma mispositioning defects (Worm #1: severe, Worm #2: mild); both populations have GFP puncta despite different number of IL neurons anterior of the midline of anterior pharyngeal bulb (dashed yellow line). L: lateral, CS: cross-section of the nerve ring. P-values from unpaired t-test are shown.

10 animals were analyzed for each panel. Representative images of L4/young adult stage animals are shown and represent maximum intensity projections of the complete Z-stack. Scale bar = 10μM.

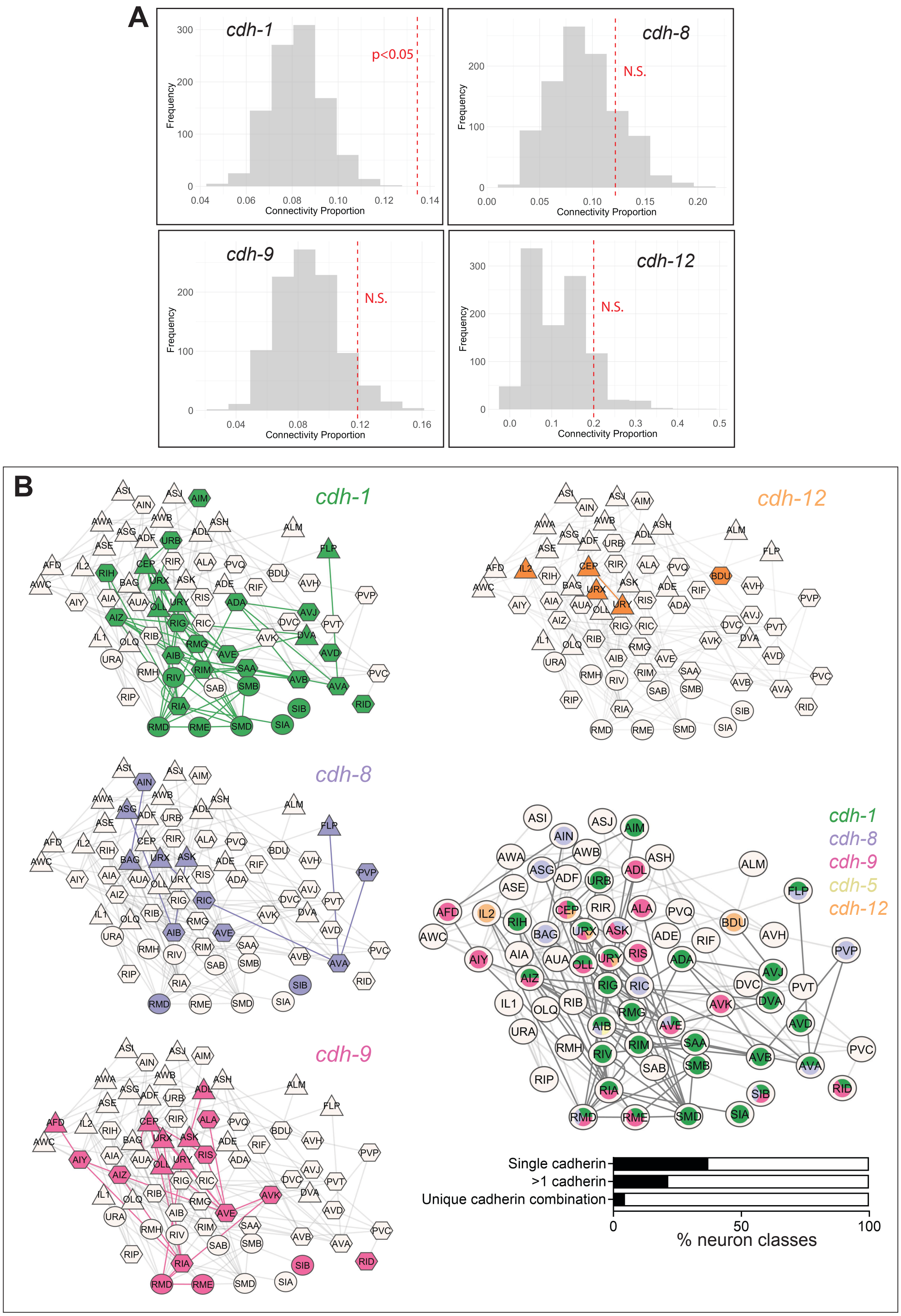

A
cdh-1
p<0.05
Frequency
Connectivity Proportion
cdh-8
N.S.
Frequency
Connectivity Proportion
cdh-9
N.S.
Frequency
Connectivity Proportion
cdh-12
N.S.
Frequency
Connectivity Proportion
B
cdh-1
cdh-12
cdh-8
cdh-9
cdh-1
cdh-8
cdh-9
cdh-5
cdh-12
Single cadherin
>1 cadherin
Unique cadherin combination
0
50
100
% neuron classes

Figure S4: Narrowly expressed cadherins are largely expressed in neurons that have conserved synapses across multiple *C. elegans* samples

(A) Comparison of observed cadherin connectivity and null distribution of connectivity for cadherins *cdh-1*, *cdh-8*, *cdh-9*, and *cdh-12*. For each cadherin, a random set of adjacent neurons - equal in number to the number of neurons expressing that cadherin - was selected. Synaptic connectivity between these neurons was shuffled over 1000 trials to derive the null distribution of connectivity proportion. The null distribution was compared to the observed connectivity proportion (red dashed line). $P<0.05$ suggests a significantly higher observed connectivity proportion than would be expected by chance, whereas $P>0.05$ (N.S.) suggests that the observed connectivity proportion is within the range of the null distribution.

(B) Expression of narrowly expressed cadherins (*cdh-1*, *cdh-9*, *cdh-8*, *cdh-12*, and *cdh-3*) overlaid on the core/conserved *C. elegans* connectome (either alone or altogether), which includes connectomes for all developmental stages and both sexes (*85*-*87*). Percentages of neurons expressing single cadherins, multiple cadherins, and unique cadherin combinations are shown.

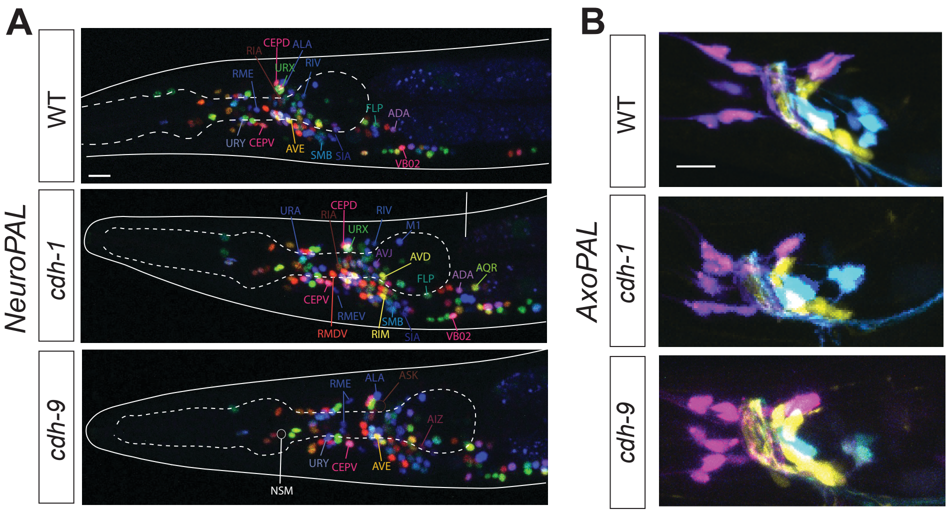

Figure S5: Broad phenotypic analysis of *cdh-1* and *cdh-9* mutants

(A) Representative images of *NeuroPAL* reporter in the background of *cdh-1(ot1034)* and *cdh-9(ot1247)* mutants. Only *cdh-1*/*cdh-9* positive neurons are labeled.

(B) Representative images of AxoPAL reporter in the background of *cdh-1(ot1034)* and *cdh-9(ot1247)* mutants.

All images are maximum intensity projections of a subset of the Z-stack. Scale bars = 10μM. 10 animals per genotype were analyzed for the NeuroPAL and AxoPAL analyses.

Figure S6

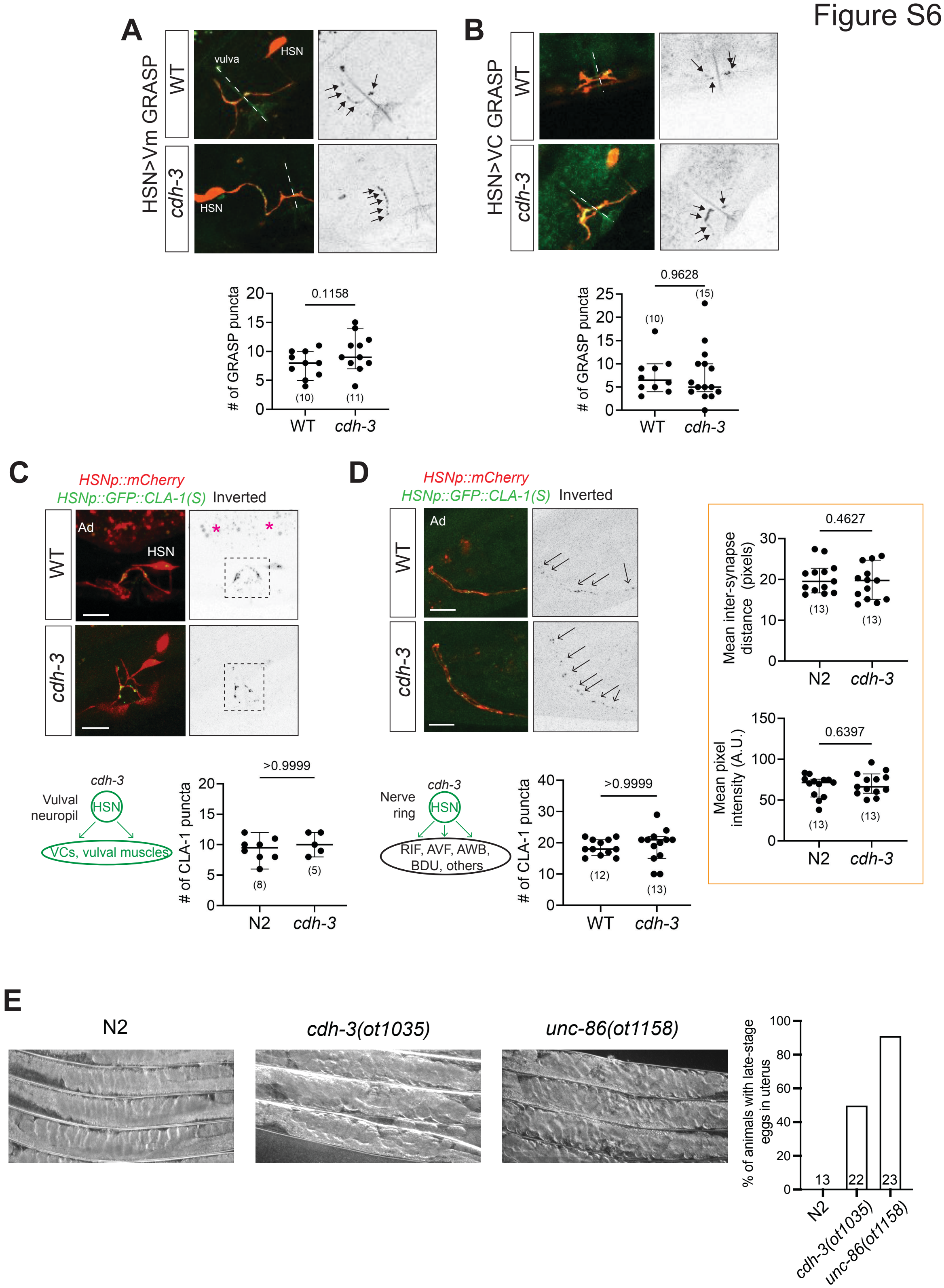
A
HSN>Vm GRASP
WT
cdh-3
vulva
HSN
# of GRASP puncta
0.1158
(10)
(11)
WT
cdh-3
B
HSN>VC GRASP
0.9628
(10)
(15)
C
HSNp::mCherry
HSNp::GFP::CLA-1(S)
Inverted
Ad
cdh-3
Vulval neuropil
VCs, vulval muscles
# of CLA-1 puncta
>0.9999
(8)
(5)
N2
D
Nerve ring
RIF, AVF, AWB, BDU, others
(12)
(13)
Mean inter-synapse distance (pixels)
0.4627
Mean pixel intensity (A.U.)
0.6397
E
N2
cdh-3(ot1035)
unc-86(ot1158)
% of animals with late-stage eggs in uterus
13
22
23

Figure S6: Phenotypic analysis of *cdh-3/Fat* mutants

(A) HSN>Vm GRASP signal is unaffected in *cdh-3(ot1035)* mutants.

(B) HSN>VC GRASP signal is unaffected in *cdh-3(ot1035)* mutants.

(C, D) HSN-specific presynaptic CLA-1::GFP puncta in the vulval region (C) and nerve ring (D) are unaffected in *cdh-3(ot1035)* mutants. Quantitative features other than puncta number – scored using WormPsyQi - are also unaffected in the nerve ring. Gut autofluorescence is marked with an asterisk.

(E) Egg-laying defects in *cdh-3(ot1035)* mutants were compared to wild-type N2 and *unc-86(ot1158)* null mutants (positive control). Late-stage eggs are embryos that have passed the comma stage (430 min of development). Eggs are normally laid at ~150 min of embryonic development.

All images are maximum intensity projections of a subset of the Z-stack. Scale bars = 10μM. In all graphs, a dot represents one worm and error bars denote median with 95% confidence interval. P-values from unpaired t-test are shown.

Figure S7

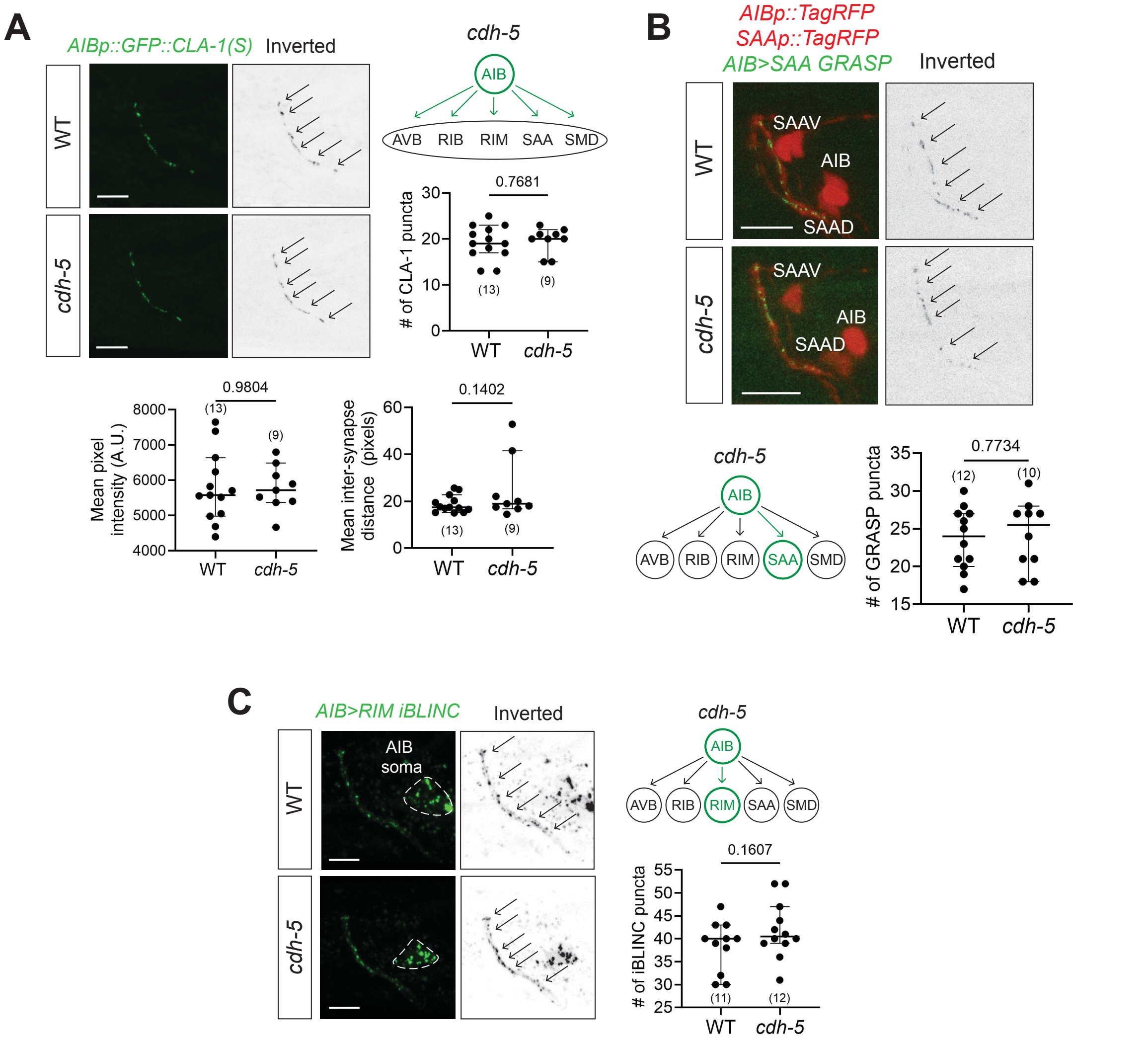

Figure S7: Chemical synapses are unaffected in *cdh-5* mutants

(A) AIB presynaptic specializations visualized with *AIBp::GFP::CLA-1* (*otIs886*) are unaffected in *cdh-5(ot1093)* null mutants. *cdh-5* is exclusively expressed in AIB neurons. No phenotype was observed in the number, mean pixel intensity, and mean inter-synaptic distance of CLA-1 puncta.

(B) AIB>SAA synapses, assessed with an NLG-1 GRASP reporter (*otEx7809*), are unaffected in *cdh-5(ot1093)* mutants. *cdh-5* is expressed only in AIB neurons.

(C) AIB>RIM synapses assessed with an iBLINC reporter (*dzIs89*) are unaffected in *cdh-5(ot1093)* mutants. *cdh-5* is expressed only in AIB neurons.

All images are maximum intensity projections of a subset of the Z-stack. Scale bars = 10μM. In all graphs, a dot represents one worm and error bars denote median with 95% confidence interval. P-values from unpaired t-test are shown.

Figure S8

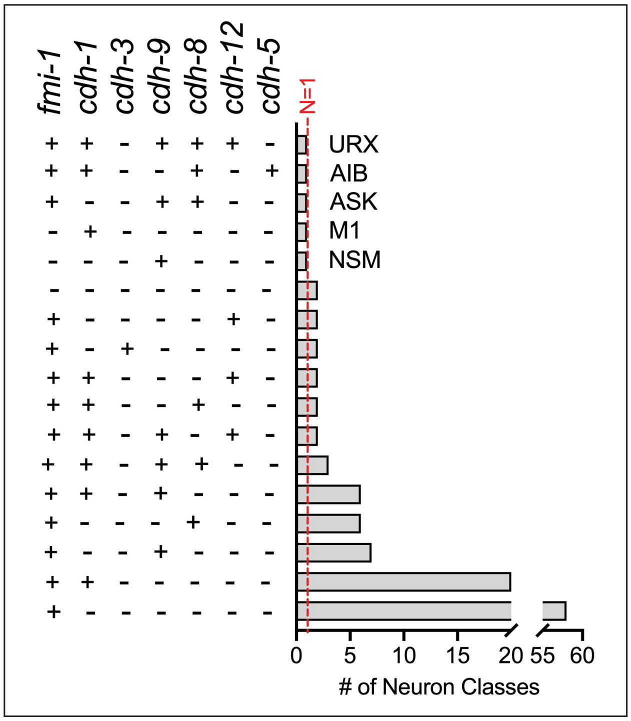

Figure S8: Distribution of combinational cadherin expression codes

Bar plot showing the distribution of combinatorial cadherin codes based on the developmental expression of cadherins *cdh-1*, *cdh-3*, *cdh-5*, *cdh-8*, *cdh-9*, *cdh-12*, and *fmi-1*. Five neurons have unique cadherin combinations (N=1, dashed line). The remaining combinations are expressed in 2 or more neurons.

Figure S9

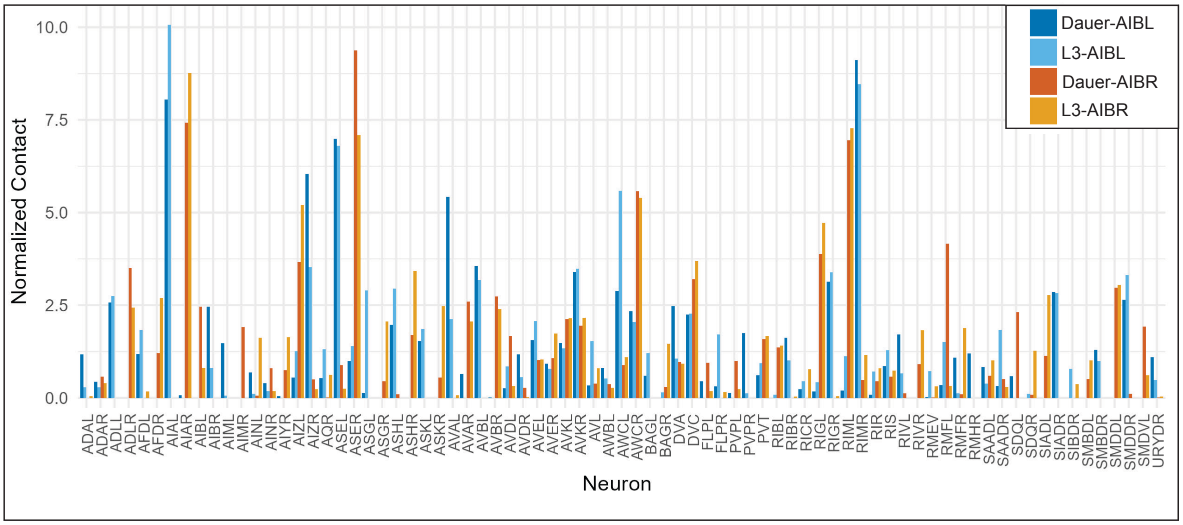

Figure S9: Comparison of neuronal contact between AIB and partner neurons in dauers vs. L3 animals

Bar plot showing EM reconstruction-based normalized contact (*86, 96*) between AIBL/R and partner neurons. For each neuron pair, dauer vs. L3 contact is plotted; bilateral neurons are separated in L vs. R to denote asymmetric changes. N=1 for both L3 and dauer samples.

Table S1: Cellular expression of cadherin reporters

Table S2: Comparison of CRISPR reporter and embryonic scRNA-sequencing cadherin expression

Table S3: Locomotory analysis of cadherin mutants

Table S4: Summary of phenotypes studied

Table S5: List of strains
